# Contribution of miRNAs, tRNAs and tRFs to Aberrant Signaling and Translation Deregulation in Lung Cancer

**DOI:** 10.3390/cancers12103056

**Published:** 2020-10-20

**Authors:** Ilias Skeparnias, Dimitrios Anastasakis, Katerina Grafanaki, George Kyriakopoulos, Panagiotis Alexopoulos, Dimitrios Dougenis, Andreas Scorilas, Christos K. Kontos, Constantinos Stathopoulos

**Affiliations:** 1 Department of Biochemistry, School of Medicine, University of Patras, 26504 Patras, Greece; iskeparnias@upatras.gr (I.S.); dimitrios.anastasakis@nih.gov (D.A.); grafanaki@upatras.gr (K.G.); g.kyriakopoulos@upnet.gr (G.K.); 2 National Institute of Musculoskeletal and Arthritis and Skin Disease, NIH, 50 South Drive, Room 1152, Bethesda, MD 20892, USA; 3 Department of Dermatology, School of Medicine, University of Patras, 26504 Patras, Greece; 4 Department of Cardiothoracic Surgery, School of Medicine and University Hospital of Patras, University of Patras, 26504 Patras, Greece; panagiotis.alexopoulos1@nhs.net (P.A.); ddougen@med.uoa.gr (D.D.); 5 Department of Cardiothoracic Surgery, St James University Hospital, Beckett Street, Leeds LS9 7TF, UK; 6 Department of Cardiothoracic Surgery, School of Medicine and Attikon University Hospital, National and Kapodistrian University of Athens, 10679 Athens, Greece; 7 Department of Biochemistry and Molecular Biology, National and Kapodistrian University of Athens, 10679 Athens, Greece; ascorilas@biol.uoa.gr (A.S.); chkontos@biol.uoa.gr (C.K.K.)

**Keywords:** tRNAs, tRFs, miRNAs, translation, signaling, lung cancer

## Abstract

**Simple Summary:**

The profiles of miRNAs, tRNA-derived fragments and tRNAs from lung cancer biopsy specimens indicate involvement of gene networks that modulate signaling and translation initiation. The current study highlights the important role of several regulatory small non-coding RNAs in aberrant signaling and translation deregulation in lung cancer.

**Abstract:**

Transcriptomics profiles of miRNAs, tRNAs or tRFs are used as biomarkers, after separate examination of several cancer cell lines, blood samples or biopsies. However, the possible contribution of all three profiles on oncogenic signaling and translation as a net regulatory effect, is under investigation. The present analysis of miRNAs and tRFs from lung cancer biopsies indicated putative targets, which belong to gene networks involved in cell proliferation, transcription and translation regulation. In addition, we observed differential expression of specific tRNAs along with several tRNA-related genes with possible involvement in carcinogenesis. Transfection of lung adenocarcinoma cells with two identified tRFs and subsequent NGS analysis indicated gene targets that mediate signaling and translation regulation. Broader analysis of all major signaling and translation factors in several biopsy specimens revealed a crosstalk between the PI3K/AKT and MAPK pathways and downstream activation of eIF4E and eEF2. Subsequent polysome profile analysis and 48S pre-initiation reconstitution experiments showed increased global translation rates and indicated that aberrant expression patterns of translation initiation factors could contribute to elevated protein synthesis. Overall, our results outline the modulatory effects that possibly correlate the expression of important regulatory non-coding RNAs with aberrant signaling and translation deregulation in lung cancer.

## 1. Introduction

In almost all cancers, miRNAs target and control the fate of several important mRNAs involved in carcinogenesis and tumor suppression [1]. Although miRNAs are the most well-characterized class of small non-coding RNAs and have been established both as regulators and biomarkers, several new RNA species have recently emerged with similar characteristics [2]. The discovery of tRNA-derived RNA fragments (tRFs), a heterogeneous group of small non-coding RNAs (14–40 nt) first detected in the urine of cancer patients, is a representative example which highlights the regulatory role of tRNAs, beyond translation [3,4]. The major types of tRFs are tRF-5, tRF-3, tRF-1, 5′ tRNA half, 3′ tRNA half and i-tRFs and they all derive either from premature or mature tRNAs after cleavage on specific sites by important ribonucleases [5]. Mammalian tRF-5 and tRF-3 types correspond to the 5′ part or the 3′ part of the tRNA, respectively. Although the biogenesis of the majority of short tRFs is still unclear, several lines of evidence suggest that Dicer is responsible for generation of certain individual tRFs, but does serve as a general mechanism of global tRF biogenesis [6,7]. tRF-5 are divided into three subtypes (a, b, and c) which vary in size (14–16 nt, 22–24 nt and 28–30 nt respectively) and derive from different cleavage sites at the D-loop (tRF-5a) or the D-stem (tRF-5b and c). tRF-3 include two subtypes, tRF-3a and b, of 18 and 22nt respectively [8]. The generation of tRF-1 type is mediated by the excision of the 3′-UUU trailers of pre-mature tRNAs by RNase Z (encoded by *ELAC*) and further trimming. In addition, specific tRFs termed tRNA-halves (or tiRNAs) are produced by angiogenin-mediated cleavage of the anticodon loop as a protective response to various stress signals [9]. Several other internal tRNA fragments, termed i-tRFs, were proposed as a separate type of tRFs and emerged from bioinformatics meta-analyses of available NGS data [10]. This type includes introns of the anticodon loop (tRF-2), part of the anticodon and variable loop (A-tRF), the variable loop and part of T stem/loop (V-tRF) and part of the D stem/loop (D-tRF) [11]. However, the enzymatic events and conditions that mediate the generation of i-tRFs as well as their possible regulatory role are unknown, with the exception of one study reporting interactions of internal parts of tRFs deriving from tRNA^Gly^, tRNA^Asp^, tRNA^Tyr^ and tRNA^Glu^ with the oncogenic protein YBX1 [12]. The presence of tRFs has been detected in many cell types and tissues, either under normal or stress conditions, suggesting a role of tRFs as post-transcriptional regulators of gene expression, as well as novel biomarkers in disease [13]. Of note, analysis of data from photoactivatable ribonucleoside-enhanced crosslinking and immunoprecipitation (PAR-CLIP) and cross-linking ligation and sequencing of hybrids (CLASH) experiments, identified tRFs loaded onto AGO proteins and experimental verification showed that tRF-5 and tRF-3 are preferably associated with AGO1, 3 and 4 (but not AGO2) proteins [6,14]. These observations not only suggest that tRFs actively participate in gene silencing via tRF-mRNA targeting mechanisms, similar to those used in miRNA-mediated gene silencing, but also expand the current view of small non-coding RNA-mediated regulation of gene expression [14,15]. Notably, several tRFs are misannotated as miRNAs, as they seem to obey the same seed rules to target mRNAs and therefore, fluctuations in their levels could mask the efficacy of both miRNAs and siRNAs that co-exist in the cell [16,17]. Several reports have provided experimental evidence of tRF-mediated tumor suppression through inhibition of either translation initiation or general translation, regulation of ribosome biogenesis and modulation of the stability of factors which promote cell proliferation, like c-MYC [9,12,18,19,20,21]. A recent bioinformatics analysis indicated statistically significant correlations between tRFs and mRNAs encoding for important translation components, like ribosomal proteins and aminoacyl-tRNA synthetases, but experimental validation was suggested as necessary to confirm the analysis [22]. Finally, only few experimentally validated tRFs with attributed oncogenic capacity have been reported, with tRF-1001 (tRF-1 type) being among the first [23,24].

The expression levels of specific tRNA species vary significantly between normal and cancer samples and aberrant transcription of tRNAs represents an important contributing factor to translation deregulation [25,26]. Such variations (i.e., overexpression of the initiator tRNA_i_^Met^) can modulate selective translation of specific mRNAs and can drive malignant transformation of normal cells, by triggering deregulation at the translation initiation level [27,28,29]. In addition, specific tRNA pools serve the codon usage during translation of important mRNAs, either during proliferation or differentiation [30]. For example, in metastatic breast cancer, specific upregulated tRNAs can enhance ribosome occupancy and translation efficiency of pro-metastatic genes enriched in their cognate codons [31]. In some cases, tRNA-mediated translation deregulation involves misacylation and differential tRNA modification pattern, which results in defective proteome integrity and genome instability [32]. As a result, defects of the translation machinery alter the expression of tumor-promoting proteins like KRAS, mTOR and MYC which, in turn, directly affect transcription of several important RNAs and drive carcinogenesis [33]. This important regulation loop is additionally controlled by specific miRNAs which keep a balance between the expression of tumor-suppressors and tumor-promoting genes, a process in which tRFs possibly contribute significantly as well [34]. Although a significant increase in the absolute levels of tRNA genes has been observed when cancer cells were compared to healthy cells, the tRNA isoacceptors composition remains unaltered [35]. Moreover, it is evident that differential expression of tRNA genes drives the abundance of tRFs and the regulation of functions that they possibly control, without changes in the levels of mature tRNAs [36]. Bioinformatics analyses have shown that tRFs exhibit tissue type-dependent lengths, tissue-specific relative abundancies and even gender- or disease subtype-dependencies [10,37]. Therefore, tRFs are seen not only as the new nexus in RNA-mediated regulation of gene expression but also as novel cancer biomarkers [38].

Several studies have shown that mutations in oncogenes result in the integral and persistent activation of the PI3K/AKT/mTOR and/or the MAPK signaling pathways, which target directly or indirectly important downstream transcription and translation factors [33]. However, mutations could explain only part of the aberrant signaling activation [39]. Downstream signaling of both pathways eventually upregulates translation rates and increase the demand for tRNAs as substrates for protein synthesis. Depending on the cell’s status, different translation programs require different tRNA pools which contribute to generation of different tRNA fragments that could in turn, regulate important signaling or translation factors in a regulatory loop that fuels cancer progression [26,28]. So far, studies that examine simultaneously and correlate the possible contribution of the transcriptomics profile of miRNAs, tRFs and tRNAs to a net effect that leads to aberrant signaling and translation deregulation, are missing. However, big data-driven studies strongly suggest that all three important classes of small non-coding RNAs are interrelated and the net effect of their abundancies could target signal transduction and translation, thus modulating the progress of pathological conditions like cancer [22,40]. Although such studies provide important insights on the regulatory networks that are involved, in most cases they lack experimental verification which, not only could have clinical significance but could also provide a detailed outline of molecular events that eventually lead to and sustain translation deregulation. 

Therefore, in the present study, we examined the expression profile of miRNAs, tRFs and tRNAs in the same set of human lung adenocarcinoma biopsy specimens. Lung cancer is the most frequently diagnosed malignancy and a leading cause of mortality, worldwide, and approximately 85–90% of all types are classified as non-small cell lung cancer (NSCLC) [41]. Our goal was to narrow down the predicted targets of the identified miRNAs and tRFs that exhibited significant change compared to normal tissue samples. In a next step, we filtered the tRFs’ list based on the criteria of significant alteration and their previously reported association with AGO proteins, after comparison with available data from CLASH experiments [14]. Using the previously described predicted targets of tRFs with statistically significant alterations we performed gene ontology (GO) analysis which showed enrichment of gene networks that regulate gene expression and translation. Two identified tRFs were used to transfect A549 lung adenocarcinoma cells and subsequent NGS analysis revealed, among others, downregulation of important genes involved in translation, which was further verified, and could contribute to carcinogenesis. Finally, we performed polysome profiling and 48S pre-initiation complex reconstitution experiments using isolated ribosomes, total mRNA and translation initiation factors from biopsy specimens. The analysis showed increased global translation rates which was attributed to the deregulated translation factors. Our observations were supplemented by an extensive expression pattern analysis of the signaling pathways and translation factors, which revealed a crosstalk between PI3K/AKT and MAPK pathways and verified that aberrant signaling leads to deregulation of important translation factors. Collectively, our study provides a comprehensive outline of events that connects the effect of important regulatory small non-coding RNAs’ function with oncogenic signaling and translation deregulation in lung cancer.

## 2. Results

### 2.1. Patterns of miRNAs and tRFs Implicated in Transcription and Translation

**Figure 1:**
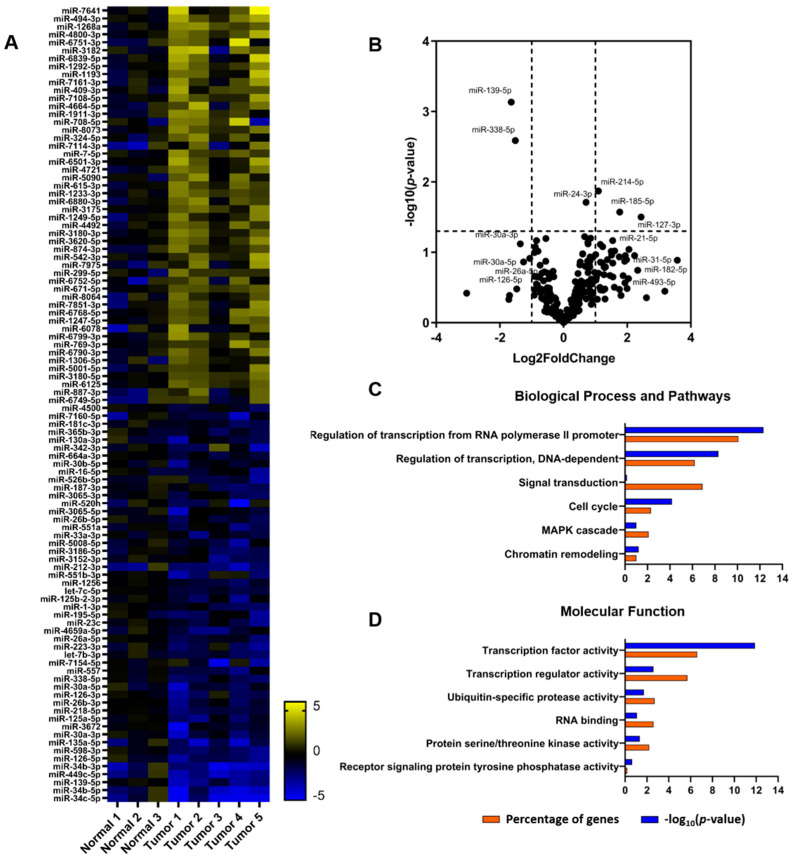
Expression profile of miRNAs in lung cancer. (**A**) Heatmap of the 100 most altered miRNAs in NSCLC. The analysis was performed in 3 normal and 5 tumor tissue specimens (Patient information is described in Table S1). The heatmap was constructed based on the log2 fold change values and normal samples are shown after normalization of individual samples versus the mean of RPM. Yellow and blue colors indicate up- and down- regulation, respectively. (**B**) Volcano plot of all miRNAs assessed in the present analysis. The volcano plot displays the relationship between fold change and significance using a scatter plot view. A higher value indicates greater significance in the y-axis and the x-axis illustrates the difference in expression levels of miRNAs. (**C**,**D**) GO enrichment analysis on the predicted targets of the statistically significant altered miRNAs. Top pathways in “Biological Process and Pathways” and “Molecular Function” are shown as percentage of genes (orange) and *p*-value (blue), respectively.

The miRNA-mediated epigenetic landscape in lung cancer has been reported as a key-indicator of gene expression regulation via translational repression [42]. To get insights on the alterations in the tumor tissue specimens compared to the normal tissues, we performed NGS and bioinformatics analysis, followed by RT-qPCR verification. We detected 845 miRNAs in total, out of which 220 were differentially expressed in tumor compared to normal tissues and were analyzed further (Figure 1A,B and Table S1). Among the miRNAs found altered, we observed statistically significant upregulation of miR-127-3p, miR-185-5p and miR-214-5p and noticeable fold change (but not statistical significance) for miR-21-5p, miR-31-5p, miR-182-5p and miR-493-5p. Among the statistically significant downregulated miRNAs were miR-139-5p and miR-338-5p. In addition, subsequent RT-qPCR verification showed downregulation of miR-26a-5p, miR-125a-5p and miR-126-5p (Figure S1). Next, we performed target prediction for the miRNAs with statistically significant alterations, followed by GO enrichment analysis. The predicted target genes were found mainly involved in transcription and translation regulator activity, cell cycle regulation and chromatin remodeling (Figure 1C,D). More specifically, several genes encoding aminoacyl-tRNA synthetases, signaling kinases and translation initiation factors were among the targets. Of note, *eIF4E* was a predicted target for miR-338-5p, which showed statistically significant change, as well as miR-31 and miR-182. In addition, miR-493-5p has been previously reported as prognostic marker for overall survival of patients with lung cancer, while miR-21 has been found to induce cell proliferation by targeting *TGFBI* [43,44]. On the other hand, predicted targets of the miR-139-5p and miR-26a-5p included, among others, genes involved in the MAPK pathway, as well as genes of cell cycle regulation. Interestingly, predicted targets of miR-30a include isoleucyl-tRNA synthetase (*IARS*) and eIF2α (*eIF2S1*), whereas *eIF2S1*, *eIF4E*, *eIF4EBP2*, *RPS6KB1* and *RICTOR* are targeted by miR-126 [45]. Overall, the GO analysis was suggestive of a link between specific miRNAs and mRNAs encoding for factors that could affect signaling and translation regulation. 

**Figure 2:**
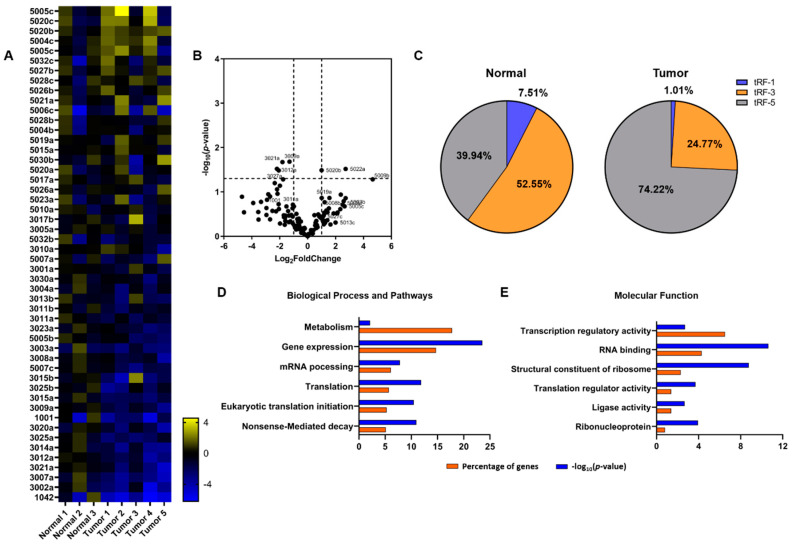
Expression profile of tRFs. (**A**) Heatmap of the 50 most altered tRFs. The analysis was performed in 3 normal and 5 tumor tissue specimens (Table S7). The heatmap was constructed based on the log2 fold change values and normal samples are shown after normalization of individual samples versus the mean of RPM. Yellow and blue colors indicate up- and down- regulation, respectively. (**B**) Volcano plot of the differentially expressed tRFs. (**C**) Distribution of tRF-1, tRF-3 and tRF-5 groups in normal and tumor specimens. (**D**,**E**) GO enrichment analysis on the predicted targets of the statistically significant altered tRFs. Top pathways in “Biological Process and Pathway” and “Molecular Function” are shown as percentage of genes (orange) and *p*-value (blue), respectively.

Previous studies featured a key-role for tRFs in cancer onset and progression, either as tumor promoters or tumor suppressors (reviewed in [38]). Although distinct signatures of tRFs are evident across several cancer types and vary in number, only few specific tRFs have been shown to modulate the expression of genes linked to tumorigenesis. Activation of oncogenes like *MYC* has been shown to modulate the expression profiles of tRFs in human lymphocytes, an observation that links translation deregulation, tRNA transcription and tRF production with early tumorigenesis [17]. After isolation of the <200 nt RNA fraction, we separated the tRNA containing fraction from the smaller RNAs (<40 nt) onto a 12% UREA-PAGE and after gel excision, libraries corresponding to each RNA pool were produced for further NGS analysis. Several available tRF databases (reviewed in [5]) are available and provide different features for bioinformatics analyses. For our analysis we used the tRF database (tRFdb) which is the first database reported and has been the basis for the meta-analysis of tRFs data from PAR-CLIP and CLASH experiments that lead to the identification of tRFs associated with AGO proteins that could play regulatory role similar to miRNAs [14]. In addition, tRFdb includes sequences of tRNA halves under either the tRF-5 or tRF-3 clustering and also tRF-1 type sequences which are absent from other databases, but have been experimentally validated and affect important biological functions [4]. The tRFdb can be searched by tRF sequence or tRF ID and therefore, we counted directly unique annotated tRF sequences in our fastq files. We identified 122 unique tRF sequences (22 tRF-1, 48 tRF-3, 52 tRF-5) with differential representation between normal and tumor samples. Among the pool of the identified tRFs we observed 19 putative tRNA-halves (based on their length; >30 nt) including tRF-5016c and tRF-5017c which derive from tRNA^Cys^_GCA_ and tRNA^Ala^_AGC_, respectively. These two tRFs bear the characteristic 5′-oligo-G motif that classifies them as putative translation initiation inhibitors (Table S2) [9,46]. Interestingly, the number of tRF-3 reads in the normal tissues are much higher than the other two tRFs types (52.55% compared to 39.94% tRF-5 and 7.51% tRF-1). In the tumor specimens, a significant increase for tRF-5 (74.22%) and significant decrease for tRF-3 (24.77%) and tRF-1 (1.01%) was observed. Analysis of the 50 most altered tRFs revealed a distinct expression pattern across the samples (Figure 2A–C). Among the statistically significant upregulated tRF-5 were tRF-5003b, tRF-5020b and tRF-5022a. In addition, tRF-5023a (deriving from tRNA-Leu-CAG) that was recently reported to promote cell proliferation and cell cycle in NSCLC, was also found upregulated [24]. On the other hand, among the statistically significant downregulated tRF-3 were tRF-3021a, tRF-3012a, tRF-3027a and tRF-3003a. We also observed downregulation of tRFs which although they exhibited fold change they were not among the statistically significant, but have been previously reported with regulatory roles like tRF-3011a (deriving again from tRNA-Leu-CAG), which is known to regulate the expression levels of the ribosomal protein RPS28 and tRF-1001 which was among the first tRFs to be associated with cancer (Figure 2A,B) [18,23]. The expression pattern of several tRFs was verified via RT-qPCR using adaptor and specific primers, as has been described previously [24] (Materials and Methods, Figure S2).

To examine the putative involvement of the tRFs from our analysis in the regulation of important gene networks, we compared our tRF list, with the previously reported list of tRFs that have been found loaded on AGO proteins [14]. We identified a significant number of tRFs (*n* = 68; 55.74%) which are putative AGO binders and could mediate post-transcriptional regulation of gene expression. The target prediction for tRFs with statistically significant alterations which was available in the literature, was used for subsequent GO enrichment analysis. The GO analysis revealed that among the putative targets of the tRFs that we identified, are genes that mediate gene expression regulation, mRNA processing and translation (Figure 2D,E and Table S3).

### 2.2. tRNA Profiling and Expression Levels of Related Genes 

**Figure 3:**
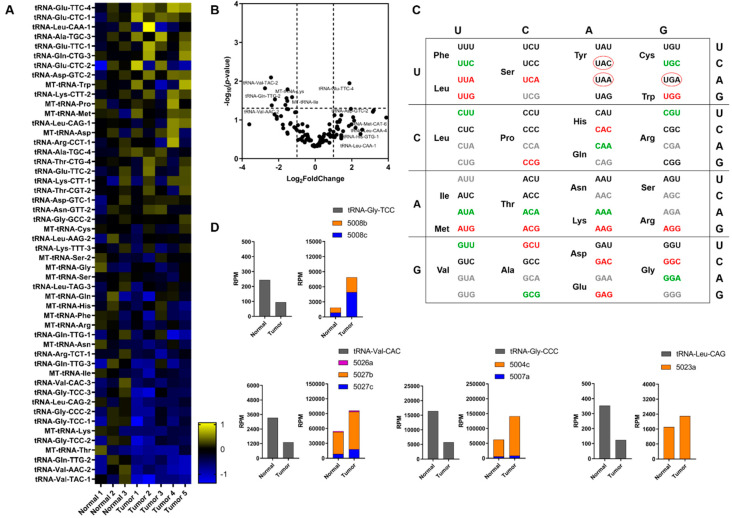
Expression profile of tRNAs. (**A**) Heatmap of the most altered tRNAs. The analysis was performed in 8 samples (5 tumor and 3 normal tissue specimens, Table S7). The heatmap was constructed based on the log2 fold change values and normal samples are shown after normalization of individual samples versus the mean of RPM. Yellow and blue colors indicate up- and down- regulation, respectively. (**B**) Volcano plot of the detected tRNAs assessed in the present analysis. (**C**) Expression pattern of tRNAs grouped in 64 codons. Red color denotes up-regulation and green color denotes down-regulation. Grey color denotes tRNAs that remain relatively unaltered and black color denotes tRNAs that were not identified in the present analysis. Stop codons are indicated in black color and red circles. (**D**) Representative downregulated tRNAs in tumor specimens and their reciprocal correlation with tRNA fragments that derive from those tRNAs.

Recent studies have linked specific tRNA expression profiles and tRFs with tumorigenesis and cancer progression, along with several genes related to tRNA biology which mediate important mechanisms [40]. In our study, we detected expression of 474 tRNAs based on the annotated sequences deposited in the Genomic tRNA database [47]. We focused on a pool of 116 tRNAs deriving from a more stringent filtering of the data which was based on reads that correspond to a minimum length of 40 nt, to ensure identification of intact tRNAs (Table S4). Our results from tumor biopsy specimens indicated statistically significant upregulation for tRNA-Glu-TTC-4. In addition, tRNA-Leu-CAA-1, tRNA-Asp-GTC-2, tRNA-His-GTG-1 and tRNA-Leu-CAA-4 were found upregulated but were not among the statistically significant (Figure 3A,B). On the other hand, we observed statistically significant downregulation of tRNA-Val-TAC-2, tRNA-Val-TAC-1, tRNA-Leu-CAG-2, tRNA-Gln-TTG-2, tRNA-Gly-CCC-2, tRNA-Gly-TCC-2, tRNA-Val-AAC-2 and mitochondrially encoded tRNA^Lys^ and tRNA^Ile^. In addition, in the same pool of tRNAs we analyzed the differential expression at the codon level. Interestingly, 25 out of 40 represented codons (~62.5%) showed alterations in their expression and 14 of them (~56%) were found upregulated (Figure 3C). Next, we investigated whether downregulation of specific tRNAs is correlated with specific tRF upregulation. After examining the downregulated tRNAs we observed noticeable reciprocal correlations between tRNA^Gly^_TCC_, tRNA^Gly^_CCC_, tRNA^Leu^_CAG_ and tRNA^Val^_CAC_ and the increased production of their cognate tRFs (Figure 3D). More specifically, we observed significant increase of tRFs 5008b and 5008c (deriving from tRNA^Gly^_TCC_), 5004c and 5007a (deriving from tRNA^Gly^_CCC_), 5023a (deriving from tRNA^Leu^_CAG_) and 5026b, 5027b and 5027c (deriving from tRNA^Val^_CAC_). 

The levels of important genes involved in tRNA transcription, maturation, transport and aminoacylation in the above-mentioned processes were measured and we observed upregulation of *SSB* which encodes the La scaffold protein that facilitates proper folding of pre-tRNAs. In addition, the expression of almost all genes encoding for the protein subunits of RNase P (the ubiquitous ribonuclease responsible for 5′ maturation of pre-tRNAs) were also found upregulated. Among those genes, *RPP14*, *RPP20*, *RPP30* and *RPP38* exhibited the most significant upregulation (Figure S3A). These results could be related with the demand of cancer cells for increased mature tRNA production to serve the elevated translation rates. In the same line, the expression of genes that encode important aminoacyl-tRNA synthetases (aaRSs) was examined. It is known that several aaRSs are correlated to angiogenesis (WARS, YARS, EPRS, RRS and IRS), immune response (YARS, KRS and RRS) and cell proliferation (KARS and MARS) [48]. In our study, we observed upregulation of *MARS* and *EPRS* and downregulation of *WARS*, *QARS* and *AIMP1* (encoding for the non-enzymatic scaffold protein p43) (Figure S3B). Our analysis showed a distinct tRNA expression profile, which could contribute to mechanisms related to carcinogenesis and cancer progression and verified that several genes related to tRNA biogenesis and aminoacylation may participate as well, as has been suggested by previous bioinformatics analyses [22,40]. 

### 2.3. Specific tRFs Can Target Translation-Related Factors

**Figure 4:**
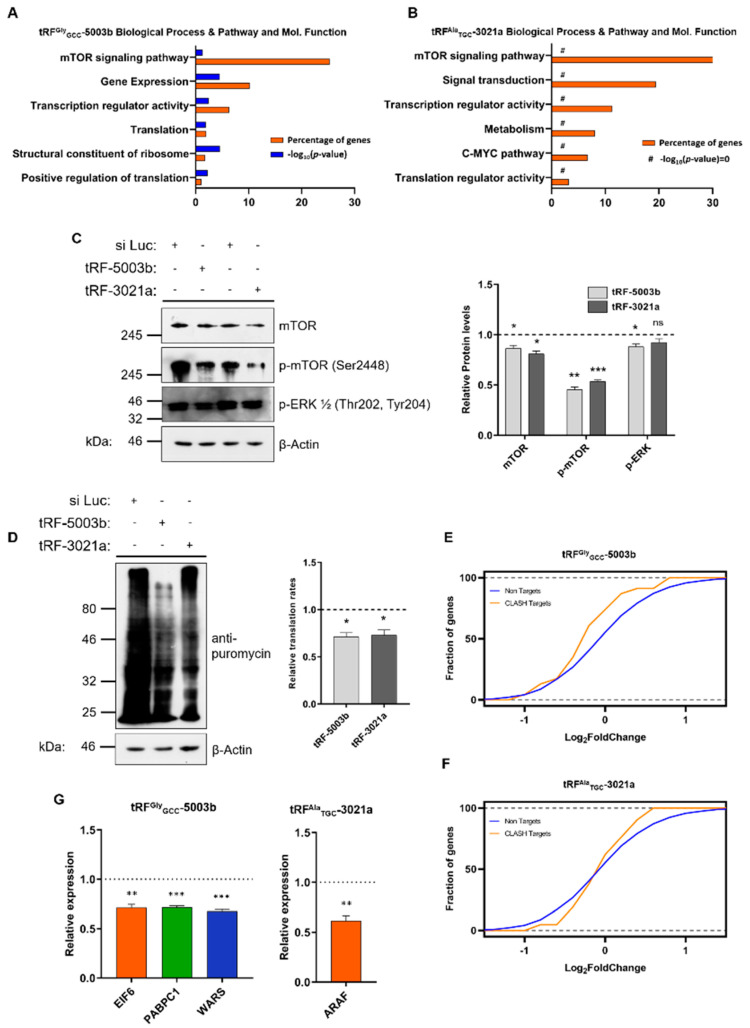
Effect of tRF^Gly^_GCC_-5003b and tRF^Ala^_TGC_-3021a on signaling and translation. (**A**,**B**) GO enrichment analysis of genes exhibiting statistically significant expression alternations after transfection of tRF^Gly^_GCC_-5003b and tRF^Ala^_TGC_-3021a in A549 lung adenocarcinoma cell line. Enriched pathways in “Biological Process & Pathway and Mol. Functions” are shown as percentage of genes (orange) and *p*-value (blue), respectively [#: −log_10_(*p*-value) = 0]. (**C**) Effects of tRF-5003b and tRF-3021a on signaling effectors and translation rates. Western blot analysis of the signaling kinases mTOR and ERK1/2 after transfection with si Luciferase (si Luc), tRF-5003b or tRF-3021a. Phosphorylated residues numbers are indicated. Quantitation of protein levels was performed after normalization to *β*-actin levels. Changes in phospho-ERK1/2 were quantified by summing the intensities of the two phosphorylated forms (ERK1 and ERK2). (**D**) Non-radioactive measurement of translation rates after assessment of the levels of puromycylated nascent peptides by Western blot. Quantitation of puromycylated peptides levels was performed after normalization to *β*-actin levels. (**E**) Cumulative distribution function (CDF) plots showing the repression of tRF^Gly^_GCC_-5003b and (**F**) tRF^Ala^_TGC_-3021a targets upon transfection of both tRFs in A549 lung adenocarcinoma cell line. Orange line represents the expression level of each high score predicted gene target in comparison to non-target genes (blue line). (**G**) RT-qPCR analysis of *EIF6*, *PABPC1*, *WARS* and *ARAF* expression after tRF-5003b and tRF-3021a transfection of A549 lung adenocarcinoma cell line respectively. Unpaired t test was used for the statistical analysis and asterisks represent *p*-values (* *p* < 0.05; ** *p* < 0.01; *** *p* < 0.001; ns, not significant).

The detected tRFs were initially sorted based on their statistical significance and then we tried to verify the levels of representative tRFs with previously reported association with AGO proteins, using RT-qPCR. We focused on tRFs which exhibited both verified altered expression and could target important genes (Figure S2 and Tables S2 and S3) and we chose tRF-3021a (deriving from either tRNA^Ala^_UGC_ or tRNA^Ala^_CGC_; downregulated) and tRF-5003b (deriving from tRNA^Gly^_GCC_; upregulated) as representatives for further experimentation. The synthetic sequence of each tRF was used to transiently transfect A549 lung adenocarcinoma cells and the transfection efficiency (almost 4-fold) was verified using RT-qPCR using adaptor and specific primers for the two tRFs (data not shown, see Materials and Methods). Subsequent NGS analysis was used to obtain the transcriptomic profile of the transfected cells. The analysis showed that both tRFs had a significant effect on the expression of several important genes. Subsequent GO enrichment analysis was performed to analyze the sets of genes with statistically significant alterations after expression of tRF-5003b or tRF-3021a. Transfection of A549 cells with tRF-5003b affected 2534 genes which are involved in networks that regulate the mTOR signaling pathway, gene expression, transcription and translation regulation and structural constituents of ribosomes (Figure 4A and Table S5). On the other hand, transfection of tRF-3021a affected 63 genes that exhibited statistically significant alterations. Again, the genes affected are involved in similar networks, including the mTOR signaling pathway, signal transduction and transcription and translation regulation. Due to the significantly smaller number of the genes that are affected in the latter case, the GO enrichment analysis didn’t provide statistical significance (Figure 4B and Table S6). To verify the possible involvement of tRF-5003b and tRF-3021a in signaling and translation regulation we examined the expression levels of active ERK and m-TOR using western blot analysis, and both were found downregulated, a result which coincides with subsequent results from the analysis of the biopsy specimens (Figure 4C). Our observations were further consolidated by measuring the relative translation rates of A549 cells transfected with either tRF, after puromycin incorporation into nascent peptides, since puromycylated peptides levels have been established to be proportional to the global translation (Figure 4D) [49]. Indeed, we observed downregulation of translation by both tRFs, a result that coincides with our GO enrichment analysis and results from subsequent analysis of the biopsy specimens. Next, we compared the gene lists obtained from the NGS analysis (Tables S5 and S6) with available CLASH data that link each tRF with putative gene targets [14]. The intersection between the two gene groups showed that the expression of specific genes is downregulated because of each tRF action (Figure 4E,F). Using RT-qPCR, we verified that major components of translation regulation like *EIF6*, *PABPC1* and *WARS* are downregulated by tRF-5003b, while the kinase *ARAF* is downregulated by tRF-3021a (Figure 4G). 

### 2.4. Aberrant Signaling Targets Important Translation Factors

**Figure 5:**
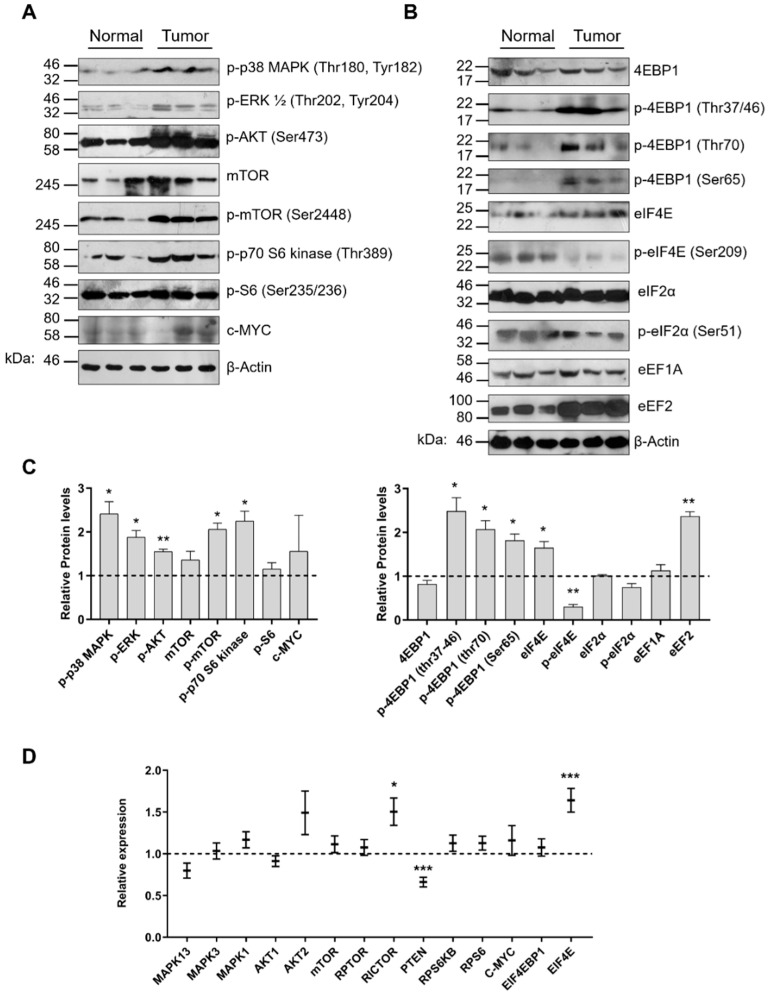
Alterations of PI3K/AKT/mTOR pathway, MAPK pathway, translation initiation and elongation factors in lung cancer. (**A**,**B**) Western blot analysis of signaling kinases, translation initiation factors, binding proteins (phosphorylated or non-phosphorylated) and translation elongation factors. Phosphorylated residues numbers are indicated. (**C**) Quantitation of protein levels after normalization to *β*-actin levels. Changes in phospho-ERK1/2 were quantified by summing the intensities of the two phosphorylated forms (ERK1 and ERK2). Asterisks represent *p*-values after unpaired t test between the relative quantity of each protein in normal and tumor specimens (* *p* < 0.05; ** *p* < 0.01; *** *p* < 0.001). (**D**) RT-qPCR analysis of genes involved in translational regulation. Expression levels analyzed in 18 tumors and 12 normal tissue specimens (Table S7). All experiments were performed in triplicates, bar graphs represent mean ± SEM (error bars) and one-way Anova test was used for the statistical analysis.

Based on the transcriptomic profile of miRNAs, tRFs and tRNAs and the fact that statistically significant miRNAs and tRFs detected in the present study have common predicted gene targets that play role in signaling pathways and translation regulation, we examined the expression and activation patterns of key components from both processes, after comparison of tumor and normal tissue specimens. The most dominant signaling pathways activated in lung cancer are the PI3K/AKT/mTOR and the MAPK pathways [41]. Both affect translation at multiple levels and their activation was also apparent in the tumor specimens that we analyzed. In all cases, the protein levels of the mTORC2-targeted p-AΚΤ (Ser473) and the RAF/MEK-targeted extracellular signal-regulated kinases p-ERK1/2 (Thr202, Thr204) were found upregulated and activated suggesting that both pathways contribute to downstream signaling (Figure 5A,C). Aberrant activation of the PI3K/AKT pathway has been reported in high prevalence in NSCLC and has been linked to cancer progression [50]. Of note, the mRNA levels of p38MAPK (*MAPK13*) and ERK1/2 (*MAPK3* and *MAPK1*) were found without significant changes, while the respective phosphorylated protein levels were upregulated (Figure 5A,C,D).

This observation coincides with previous immunohistochemical analyses suggesting that overactivation rather than overexpression of these kinases is responsible for aberrant signaling [51]. Although the mTOR kinase was found unaltered both at the transcriptional and protein level, the levels of activated p-mTOR (Ser2448) were found more than 2-fold higher in tumor specimens compared to the normal tissue (Figure 5A,C,D). Excessive mTOR activation signifies the contribution of both mTORC1 and mTORC2 complexes in downstream targeting and possible stimulatory effects on all RNA polymerase transcription initiation complexes [52]. Interestingly, the expression level of *PTEN*, a classical tumor suppressor and the most important PI3K pathway homeostatic regulator, was found slightly downregulated and in reciprocal correlation with the levels of *RICTOR*, an important scaffold of mTORC2 complex (Figure 5D). Therefore, although activation of mTORC1 leads to p70S6K activation, the latter does not affect the levels of phosphorylated S6 protein which is a constituent of the 40S ribosomal subunit (Figure 5A,C,D). Interestingly, MYC expression profile showed no significant alterations both at mRNA and protein levels, an observation that possibly suggests the existence of a more complex regulatory mechanism (Figure 5A,C,D). 

Translation initiation is regulated via 4E binding proteins (4E-BPs) which upon phosphorylation, release eIF4E which becomes available for cap binding and interaction with eIF4G. Phosphorylation of 4E-BP1, the most abundant among 4E-BPs, occurs hierarchically at threonine 37/46, threonine 70, and finally at serine 65 which promotes dissociation of 4E-BP1 from eIF4E [53]. In our study, the levels of 4E-BP1 were unaffected, whereas the levels of all phosphorylated 4E-BP1 forms were found to be significantly higher (Figure 5B,C). On the other hand, although the mRNA and protein levels of eIF4E were found moderately upregulated, phosphorylation of eIF4E (p-eIF4E at Ser209) was decreased (Figure 5B–D). This observation is in agreement with previous studies suggesting that phosphorylation of eIF4E is not a requirement for protein synthesis activation [54]. In addition, we examined the possible involvement of eIF2α, an important subunit of the heterotrimeric ternary complex (TC) which recruits the initiator Met-tRNA_i_^Met^ and binds the 40S ribosomal subunit to form the 43S pre-initiation complex. In many cancers, eIF2α subunit is usually overexpressed, providing a stimulus that leads to increased rates of protein synthesis [55]. Although our analysis did not detect differences in eIF2α expression, the levels of p-eIF2α (Ser51) tended to be lower (Figure 5B,C). Phosphorylation of eIF2α at serine 51 is a major cell growth checkpoint which blocks translation initiation via inhibition of the GTP exchange factor eIF2B and has been correlated with a global reduction of protein synthesis [56]. Finally, to investigate any possible alterations that are related to translation elongation and tRNA utilization we examined the levels of eEF1A and eEF2. Although eEF1A is a phosphorylation target by several kinases, including p70S6 kinase and eEF2 upregulation has been described for ovarian, gastric, and colon cancers, their role in NSCLC has not been addressed adequately [57]. In the present study, eEF1A was unaffected, whereas eEF2 was found almost three-fold upregulated (Figure 5B,C). 

### 2.5. Lung Cancer Specimens Exhibit Elevated Global Translation Rates 

**Figure 6:**
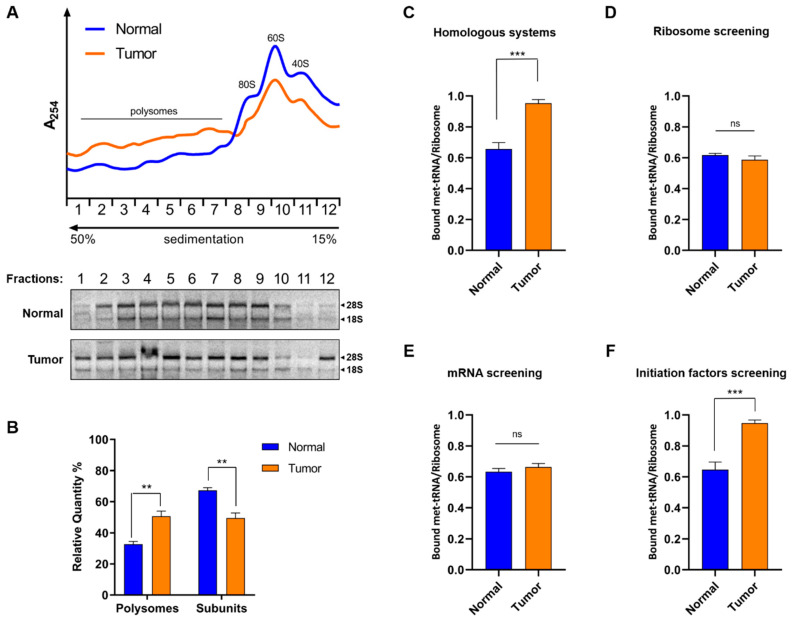
Determination of translational efficiency in lung cancer. (**A**) Polysome profiling. Ribosomal particles isolated from either normal (blue line) or tumor (orange line) lung tissue specimens were homogenized in the presence of CHX and aliquots of the S30 fraction were analyzed on 15–50% sucrose gradients. The peaks corresponding to ribosomal subunits (40S, 60S and 80S) and the polysomes tail are indicated. (**B**) Relative quantity (%) of each peak corresponding to polysomes or free ribosomal subunits between normal (blue) and tumor (orange) specimens. (**C**) Reconstitution of 48S initiation ribosomal complex in the presence of initiator [^3^H]Met-tRNA_i_^Met^, endogenous 40S ribosomal subunits, crude translation factors and total mRNA. All components were isolated from the same tissue specimens, either normal (blue) or tumor (orange). (**D**) Assay of 40S ribosomal subunits from normal (blue) or tumor (orange) specimens, in the presence of translation factors, mRNA and initiator [^3^H]Met-tRNA_i_^Met^ from normal tissue specimens. (**E**) Assay of mRNA from normal (blue) or tumor (orange) specimens in the presence of crude translation factors, 40S ribosomal subunits and initiator [^3^H]Met-tRNA_i_^Met^ from normal tissues. (**F**) Assay of crude translation initiation factors from normal (blue) or tumor (orange) specimens in the presence of 40S ribosomal subunits, mRNA and initiator [^3^H]Met-tRNA_i_^Met^ from normal tissues. All components tested, were isolated from the same specimen and experiments were performed in triplicates. Asterisks represent *p*-values after unpaired t test accomplishment between normal and tumor specimens (** *p* < 0.01; *** *p* < 0.001; ns, not significant).

To get more insights on the global translation rates of both tumor and normal tissue specimens, we evaluated the distribution of ribosomal particles after sucrose gradient centrifugation. All samples were treated with cycloheximide (CHX) which is known to prevent elongation and ribosomal run-off. The analysis of both normal (Figure 6A blue line) and tumor specimens (Figure 6A orange line) showed expected and canonical distribution patterns of ribosomal particles with distinct 40S and 60S ribosomal peaks, as well as 80S monosome and polysome peaks. Nevertheless, the quantity of polysomes from normal specimens was reduced compared to those in tumors (Figure 6B). Moreover, no signs of abnormal termination were observed, such as appearance of unusual peaks at the heavy fractions of the gradient. Therefore, the polysome profile analysis suggests that translation is stimulated in tumor specimens, presumably at the initiation and elongation phases.

Next, we examined the formation of the 48S ribosomal complex, which is the rate-limiting step of translation initiation, using a homologous system consisting of endogenous 40S ribosomal subunits, crude translation factors, endogenous total mRNA and initiator [^3^H]Met-tRNA_i_^Met^ from either normal or tumor tissue extracts. Complex formation reached approximately saturation levels (bound [^3^H]Met-tRNA_i_^Met^/ribosome was ~1) after 30 min incubation of the reaction mixture at 25 °C, with the components of the cell-free system deriving from tumor tissues (Figure 6C). However, when the same components were obtained from normal specimens, the saturation levels were calculated approximately 30% lower, supporting our previous observations which indicated higher translation efficiency in the tumor specimens. This difference can be attributed either to changes in the structure and function of the participating 40S ribosomal subunits (Figure 6D), the quality and quantity of total mRNA tested (Figure 6E) or due to changes in the participating translation initiation factors (Figure 6F). Therefore, we tested the efficiency of translation initiation complex formation by replacing each one of the above factors. Interestingly, a significant increase (~30%) in the 48S complex formation was observed, only when we tested the translation initiation factors from tumor specimens, in the presence of ribosomes and total mRNA from normal extracts (Figure 6F). This increase is consistent with the results obtained initially from the saturation experiments (Figure 6C) and strongly suggest that translation in tumor specimens is upregulated due to differences among the translation initiation factors. A diagram which illustrates the experimental procedure is presented in Supplementary Material section (Figure S4). Finally, no differences were detected either in the efficiency of initiation step or in the peptidyl-transferase activity between extracts from tumor and normal specimens (Figure S5A,B). 

## 3. Discussion

In the present study, we provide a comprehensive outline of events that link the expression patterns of miRNAs, tRFs and tRNAs with aberrant signaling and deregulated translation initiation, in lung cancer biopsy specimens (Figure S6). So far, information on miRNAs that play role in lung cancer came mainly from studies of circulating miRNAs and only two reports exist in the literature that describe miRNA profiles from tissue specimens [42,58]. Our results suggest that miRNA-mediated regulation in the tumor biopsies affects, among others, signaling and translation regulation, as has been previously suggested and this effect could impact tumor progression [58]. On the other hand, the analysis of tRFs as putative miRNA-like regulators followed by extensive experimental validation, showed that tRFs could represent novel and key-modulators of molecular events during carcinogenesis and cancer progression. A novel finding of the present study is the identification of tRF-5003b and tRF-3021a as potential regulators of important translation factors, like *EIF6, PABPC1*, *WARS* which have been previously associated with cancer progression and *ARAF* which is an important serine/threonine kinase member of the tumor promoting MAPK cascade [48,59,60,61]. More specifically, *EIF6* is responsible for the maturation of 60S subunit and has been found upregulated in NSCLC and correlated with shorter overall patient survival [59]. Accordingly, PABPC1 is an RNA-binding member of the eIF4F complex responsible for the circularization of the mRNA before binding of the ribosomal subunits and has been found upregulated in lung cancer and in interaction with AGO2, which plays a key role in miRNA-mediated gene silencing [60,62]. Finally, WARS has been correlated with angiogenesis and was found downregulated in the present study [48]. Therefore, the observed upregulation of tRF-5003b could represent a possible tumor suppressor response mechanism. On the other hand, *ARAF* is a known proto-oncogene and member of the MAPK cascade which is frequently mutated in lung adenocarcinoma and its inhibition is a promising therapeutic target and thus its targeting by tRF-3021a could possibly be associated with cancer progression [61]. 

Although tRNA overexpression is known to play role in tumorigenesis, the number of available reports regarding tRNA expression profiles in lung cancer tissue specimens is limited. A recent report using tRNA microarrays identified tRNA candidates for prognostic score prediction of survival and, a recent extensive bioinformatics analysis based on data from the Cancer Genome Atlas revealed alterations in tRNAs expression across 31 cancer types, including lung cancer. Both studies suggested that tRNA expression profiles could provide insights on translation deregulation and could be further exploited as prognostic markers. In the present study, we verified the differential expression of several tRNAs, some of which have been identified previously in datasets from other cancers [40,63]. Overexpression of specific tRNAs could serve to sense amino acids availability in rapidly proliferating cells and could indirectly enhance translation rates. One of the most downregulated nuclear-encoded tRNAs (tRNA-Leu-CAG-2) is correlated in our study with the overexpression of tRF-5023a (also known as tRF-Leu-CAG; a tRF-5 fragment) which was recently shown to induce cell proliferation and cell cycle progression in NSCLC [24]. Of note, differential expression of isoacceptor tRNAs (Figure 3C) coincides with previous studies, as in the case of tRNA^Thr^_ACA_, which is downregulated and correlates with lower survival rates across different cancer types [63]. Codon usage analysis could be beneficial to identify novel prognostic markers based on the expression levels of the corresponding tRNAs in lung cancer. Interestingly, MYC which is a major regulator of tRNA and rRNA transcription did not exhibit statistically significant alterations among the specimens examined. In addition, we observed differential expression of several genes implicated in tRNA transcription, maturation, transport and aminoacylation. Several important studies have highlighted the aberrant expression of specific synthetases or synthetase-like proteins in cancer. For example, increased levels of MARS, IRS and EPRS have been reported in several cancer types. In the present we focused mainly on aminoacyl-tRNA synthetases that participate in the multi-synthetase complex (MSC), some of which has been shown to detach from it and play signaling roles. Our data indicated downregulation of the connective protein *AIMP1*, *QARS* and *KARS*, while *MARS* and *EPRS* were found upregulated in tumor specimens. AIMP1 is responsible for interaction with RARS and QARS in the MSC, thus its downregulation could lead to release of the latter from the complex to inhibit apoptosis through interaction with ASK1, an important kinase and regulator of apoptosis, as has been reported earlier [64,65]. Taken together, our results coincide with the role of AIMP1 in the activation of JNK which can induce caspase 3, thus stimulating the apoptosis of endothelial cells [66]. Moreover, it has been shown that systemic injection of AIMP1 in mouse xenograft models can inhibit tumorigenesis [67]. Based on the previous, downregulation of QARS and AIMP1 could lead to inhibition of apoptosis, which is a hallmark of cancer. 

Our results suggest that the majority of all three classes of important non-coding RNAs that exhibited differential expression in the present study contribute to either overactivation of signaling effectors or deregulation of translation. Although it is indicated by the current and previous studies, whether this is the net result of all modulatory effects remains to be further experimentally investigated in-depth. The impact of each non-coding RNA class on gene expression regulation of each component could vary depending on the conditions. Of note, a recent report showing that RNA pol II can interfere with tRNA genes transcription by RNA pol III perplexes further the already complex regulatory network and indicates that elongating pol II represses pol III activity. These observations suggest the existence of a sensory mechanism (and possibly synchronization) that controls mRNA over tRNA transcription, under stress conditions such as serum starvation [68]. In the present study we examined and verified the activation and expression pattern of major signaling pathways that target translation factors. Our results showed that although the PI3K/AKT/mTOR axis is the major activated signaling pathway, interestingly p38MAPK is also significantly activated [51]. The RAS-dependent ERK1/2 kinases were found activated in agreement with a previous report [69]. Downstream targeting by ERK1/2 includes phosphorylation of eIF4B or eIF4E, recruitment of MYC, phosphorylation of 4E-BPs and mTOR activation [70]. On the other hand, the AKT1/2 was also found activated (Ser473) similarly to the only previous report which suggests that AKT activation is a frequent event during early lung tumorigenesis [71]. AKT phosphorylation is induced by mTORC2, a nodal stress sensor. In addition, AKT represents a key mediator and a cross-talk checkpoint between the two signaling pathways, which activates mTORC1 [72]. Our results suggest that both MAPK and PI3K/AKT pathways contribute to the aberrant downstream signaling, with the latter being more prominent. Both pathways converge to downstream regulation of mTORC1, which remains unaffected at the transcriptional level (based on the expression of *RAPTOR*). In contrast, total mTOR is highly activated and is responsible for the observed activation of p70S6 kinase. Although this activation usually leads to activation of ribosomal protein S6, we did not observe either upregulation or phosphorylation of S6, in agreement with a previous report [73]. Finally, upregulation of eEF2 can possibly be correlated with phosphorylation of eEF2 kinase by p70S6K, which leads to its inactivation and allows eEF2 to remain activated and promote translation elongation [74].

Cap-dependent translation initiation requires the recognition of the 5′cap by eIF4E. Considering that eIF4E recruitment to the cap structure is the rate–limiting step for translation initiation and that eIF4E is the least abundant among the translation initiation factors, regulation of its expression profoundly affects translation initiation efficiency [75]. The availability of eIF4E depends on its association with 4E binding proteins (4E-BPs), which release eIF4E upon phosphorylation. The events are promoted by mTORC1 which phosphorylates 4E-BP1, although the expression of 4E-BP1 was unaffected. In our study, 4E-BP1 phosphorylation occurs in all cases, as expected (Thr37/46, Thr70 and Ser 65). Phosphorylated 4E-BP1 dissociates from eIF4E and cannot longer serve as translation repressor. Although eIF4E was found significantly overexpressed, its phosphorylated form (Ser209) was reduced, an interesting and somewhat unexpected finding. Previous reports have indicated that activation of protein synthesis is independent of eIF4E phosphorylation which is not required for effective binding to eIF4G. Simple overexpression of eIF4E is sufficient to induce malignant transformation of fibroblasts and primary epithelial cells and to induce tumorigenesis, an observation that coincides with our results [50]. Moreover, in some types of cancer, including lung cancer, phosphorylated eIF4E levels were found significantly elevated only in early-stage but not in late stage tumors [76]. Of note, the expression of the main aa-tRNA-carrying factors eEF1A and eIF2α was not significantly altered. Finally, eEF2, a major elongation factor that promotes the GTP-dependent translocation of the ribosome was found upregulated, suggesting the existence of overactive elongating ribosomes, as also shown by our polysome profile analysis. The translation deregulation which occurs mainly through phosphorylation of 4E-BPs, overexpression of eIF4E and possibly through positive regulation of eEF2 (and possibly eIF4B) could promote the swift assembly of the eIF4G scaffold, the fast circularization of mRNAs and the stimulation of translating polysomes.

## 4. Materials and Methods

### 4.1. Tumor Specimens and Cell Lines

Lung adenocarcinoma biopsy specimens were obtained by the Department of Cardiothoracic surgery at the General University Hospital of Patras. The specimens were surgically removed and adjacent, phenomenally healthy tissue, was also selected at least 5 cm away from the edge of the corresponding tumors. Each sample was dissected, and one piece was subjected to routine histological or immunohistochemical classification using the revised International System for Staging Lung Cancer [77]. The remaining sample was immediately frozen at −80 °C. Prior to any experimental procedure, total RNA was extracted from each specimen and quality was assessed using a 2100 Agilent bioanalyzer. NGS analysis was performed in five tumor biopsy specimens and three normal adjacent tissue specimens (see Table S7 for details on specimens). For RT-qPCR verification of miRNAs and tRFs, total RNA from the same five tumor and three normal tissues was used. For gene expression analysis using RT-qPCR, all available tumor (*n* = 18) and normal (*n* = 12) specimens were used for analysis. For Western blot analysis, three representative tumor and three representative normal tissue specimens were analyzed (see Table S7). The A549 lung adenocarcinoma cell line was obtained from ATCC and the cells were maintained in DMEM supplemented with 10% FBS and kept at 37 °C in a humidified incubator with 5% CO_2_. The study was reviewed and approved by the Ethics Committee of the General University Hospital of Patras (Approval number: 40/10-1-2017), following the directions of the Declaration of Helsinki. Patients receiving chemotherapy or radiotherapy were excluded to avoid possible masking effect. Patient information is described in Table S7.

### 4.2. Real-Time PCR and Western Blot Analyses

Frozen tissues (~0.1 g) were immediately lysed using the PureLink miRNA Isolation Kit (Ambion, Austin, TX, USA) to isolate RNA fractions and DNA was removed using DNase I (New England Biolabs, Ipswich, MA, USA). The RNA yield and purity were determined by measuring absorbance at 260 nm/280 nm on a Quawell micro volume spectrophotometer Q3000 (Quawell Technology, Inc., San Francisco, CA, USA) and the size distribution quantity and quality were assessed using the Nano RNA Chip on an Agilent 2100 bioanalyzer. Long RNA fraction (>200 nt) was reverse transcribed using SuperScript II (Invitrogen, Waltham, MA, USA) and oligodT primer according to the manufacturer’s protocol. Separation of tRFs prior to RT-qPCR was based on selection of sizes (<40 nt) on a 12% UREA-PAGE and subsequent verification using RNA 6000 Nano chip on a Bioanalyzer (Agilent). Small RNA fractions (<200 nt) were then polyadenylated using *E. coli* Poly(A) Polymerase (New England Biolabs) and reverse transcribed using SuperScript II (Invitrogen, Waltham, MA, USA) and oligodT adapter primer, which contains the sequence recognized by the reverse primer (outer primer) for detection of miRNAs and tRFs. qPCR reactions were performed using KAPA SYBR FAST qPCR Kit (Kapabiosystems, Cape Town, Western Cape, South Africa) using 50 ng cDNA as template. Normalization of gene expression was performed based on the expression levels of *ACTB.* miR-103 was used to normalize the expression levels of small non-coding RNAs as it has been reported in previous studies [78,79]. Reactions were set up in 96-well plates and performed on an MX3000P qPCR system (Agilent, Santa Clara, CA, USA). The Ct values were extracted and further analyzed using the 2^-ΔΔCT^ method. All experiments were performed in triplicates and the primers sequences are shown in Tables S8–S10. 

For western blot analyses, tissue specimens (~0.1 g) were homogenized at 4 °C in the presence of 1ml lysis buffer [25 mM Hepes-KOH, pH 7.5, 12 mM KCl, 1 mM EDTA, 1 mM Dithiothreitol, 1% NP-40, 1% (*v/v*) Protease Inhibitor Cocktail (Sigma-Aldrich, St. Louis, MO, USA), and 1% (*v/v*) Phosphatase Inhibitor Cocktail (Sigma-Aldrich, St. Louis, MO, USA)]. The lysate was centrifuged at 15,000× *g* for 30 min at 4 °C and samples (40 μg total protein) were separated by SDS-PAGE, transferred to Immobilon-P PVDF membranes (Millipore, Massachusetts, MA, USA) and visualized after exposure to X-ray film (UltraCruz Autoradiography Film, No. sc-201696, Santa Cruz Biotechnology, Dallas, TX, USA). The band intensity measurements of each experiment were analyzed using the Image Lab software (Bio-Rad, version 6.1, Berkeley, CA, USA). The protein levels were normalized against *β*-actin levels in each separate set of experiments. The antibodies were used according to the manufacturer’s instructions and are shown in Table S11 and uncropped images were provided as supplementary material (Figures S7–S32).

### 4.3. Next Generation Sequencing and Bioinformatics Analysis

Small non-coding RNAs from tumor and normal specimens were isolated using the mirVana miRNA Isolation Kit (Ambion, Austin, TX, USA). cDNA libraries were prepared using the Ion Total RNA-Seq Kit v2 protocol for small RNA sequencing (Ion Torrent, Invitrogen, Waltham, MA, USA). Yield and size distribution of the cDNA libraries were assessed using the Agilent 2100 Bioanalyzer DNA1000 chip. Sequencing was performed using an IonTorrent 318 chip and the Ion PGM 200 sequencing kit on an IonTorrent PGM platform. In total, five NSCLC specimens and three control samples were analyzed. Sequences were mapped to an artificial genome consisting of mature human miRNAs from the miRBase and counted using BEDTools coverage (v2.29.2) [80]. The tRNA sequences were mapped to an artificial tRNA genome consisting of unique sequences of hg38 tRNAs using Bowtie (v.1.2.3) and further counted using BEDTools coverage (v2.29.2) [81,82]. In addition, we counted directly tRF sequences in the fastq files using tRF database which includes publicly available data (tRFdb; http://genome.bioch.virginia.edu/trfdb/). RPM values for each molecule were calculated from the corresponding reads. The average across the replicates between normal and tumor samples was used to measure the fold-change by dividing the corresponding mean values. Unpaired *t* test was used for the statistical analysis. The detected miRNAs and tRFs were further analyzed for their ability to act in putative gene targets through TargetScan 7.2 and based on available data from CLASH analyses, respectively [14,83]. FunRich 3.1.3 was used for Gene Ontology enrichment and pathway analysis [84]. Transcriptomic analysis was performed after transient transfection of A549 lung adenocarcinoma cells using the two synthetic tRFs tRF^Ala^_TGC_-3021a or tRF^Gly^_GCC_-5003b (Eurofins Genomics, Table S12). Isolation of total RNA using the PureLink RNA Mini kit (Invitrogen, Waltham, MA, USA) and preparation of cDNA libraries using the NEBNext Ultra II Directional RNA Library Prep Kit for Illumina (New England Biolabs, Ipswich, MA, USA). NGS was performed on an Illumina HiSeq3000 platform. The scripts used for our analysis are available upon request and all the raw and summarized data regarding expression profiles of miRNAs, tRNAs, tRFs and mRNAs from all the performed experiments are available at the GEO Repository under the Accession Number GSE143694.

### 4.4. Non-Radioactive Measurement of Translation Rates

Transient transfection of A549 lung adenocarcinoma cells using the two synthetic tRFs tRF^Ala^_TGC_-3021a or tRF^Gly^_GCC_-5003b (Eurofins Genomics, Table S12) was followed by incubation of cells with 1 μM puromycin for 10 min. Cells were then lysed in lysis buffer [25 mM Hepes-KOH, pH 7.5, 12 mM KCl, 1 mM EDTA, 1 mM Dithiothreitol, 1% NP-40, 1% (*v/v*) Protease Inhibitor Cocktail (Sigma-Aldrich), and 1% (*v/v*) Phosphatase Inhibitor Cocktail (Sigma-Aldrich, St. Louis, MO, USA)]. The lysate was centrifuged at 15,000× *g* for 30 min at 4 °C and samples (40 μg total protein) were separated by SDS-PAGE, transferred to Immobilon-P PVDF membranes (Millipore, Massachusetts, MA, USA) and visualized after exposure to X-ray film (UltraCruz Autoradiography Film, No. sc-201696, Santa Cruz Biotechnology, Dallas, TX, USA). Band intensities were quantified using the Image Lab software (Bio-Rad). The puromycylated peptides levels were normalized against *β*-actin levels in each separate set of experiments. The antibodies were used according to the manufacturer’s instructions and are shown in Table S11. 

### 4.5. Sedimentation Profile of Ribosomal Particles and Translation Efficiency Measurements

Tissue specimens (~0.1 g) were supplemented with 3 mL of buffer A [50 mM Tris-HCl, pH 7.5, 150 mM NH_4_Cl, 10 mM (CH_3_COO)_2_Mg, 0.5 mM EDTA, 58 μg/mL phenylmethylsulfonyl fluoride (PMSF), 250 mM sucrose, 200 μM cycloheximide (CHX), and 6 mM *β*-mercaptoethanol], followed by homogenization at 4 °C. The homogenate was adjusted to 0.5% deoxycholate and centrifuged at 13,000× *g* for 20 min at 4 °C, followed by a second centrifugation at 30,000× *g* for 1h at the same temperature. Approximately 20 A_260_ units of the final supernatant (S30 fraction) were loaded on a 15–50% linear sucrose gradient in buffer A and centrifuged in an SW41 rotor at 37,000× rpm for 4 h at 4 °C and analyzed by optical scanning at 254 nm. Fractions were analyzed on a 2% agarose gel (Figure 6). Uncropped images were provided as supplementary material (Figures S33 and S34). For measuring the activity of ribosomes, total RNA was isolated from normal tissue specimens, using TRIzol RNA isolation reagent (Invitrogen, Waltham, MA, USA) followed by mRNA isolation, using the Oligotex mRNA kit (Qiagen, Hilden, Germany). [^3^H]Met-tRNA_i_^Met^ or [^3^H]Phe-tRNA^Phe^ were prepared, using yeast tRNA_i_^Met^ or tRNA^Phe^ (Sigma-Aldrich, St. Louis, MO, USA), radioactive methionine ([^3^H]Met) or phenylalanine ([^3^H]Phe) (PerkinElmer, Inc.) and partially purified yeast extracts containing the full set of aminoacyl-tRNA synthetases. [^3^H]Phe-tRNA^Phe^ was then acetylated (Ac[^3^ H]Phe-tRNA^Phe^) using acetic acid anhydride. Crude translation factors, highly active run-off 80S ribosomes and free ribosomal subunits (40S and 60S) from tumor specimens were also prepared. Post-translocation complex of poly(U)-programmed ribosomes from lung cancer, carrying tRNA^Phe^ at the E-site and Ac[^3^H]Phe-tRNA^Phe^ at the P-site (complex C) were prepared, as described previously [85]. For the calculation of the ribosome concentrations, we adopted the following relationships: one A_260_ units equivalent to 60 pmol 40S ribosomal subunits, 30 pmol 60S ribosomal subunits, or 20 pmol 80S ribosomes [86].

### 4.6. Supplementary Methods

#### Ribosomal Complex and Peptide-Bond Formation Assays

40S ribosomal subunits (0.5 A_260_ units) were added in 50 μL of 50 mM Tris-HCl buffer, pH 7.5, containing 1 mM ATP, 0.4 mM GTP, 2 μg mRNA isolated from tumor specimens, 3 A_260_ units [^3^H]Met-tRNA_i_^Met^, 20 μg protein of crude translation factors, 6 mM *β*-mercaptoethanol and optimized amounts of metal ions and polyamines [100 mM CH_3_COOK, 5 mM (CH_3_COO)_2_Mg, 0.6 mM spermine, and 0.8 mM spermidine]. After incubation at 25 °C, the amount of the resultant 48S ribosomal complex was measured by nitrocellulose filtration in various time points. The calculated values represent measurements when binding reached saturation levels. Poly(U)-programmed 80S ribosomes (0.5 A_260_ units) isolated from tumor specimens, pre-filled in the P-site with tRNA^Phe^ were incubated for 30 min at 25 °C with 1.5 A_260_ units Ac[^3^H]Phe-tRNA^Phe^ in 50 μL of 50 mM Tris-HCl buffer, pH 7.5, containing 0.4 mM GTP, 20 μg protein of crude translation factors, 6 mM *β*-mercaptoethanol, and optimized ion concentration. The amount of bound Ac[^3^H]Phe-tRNA^Phe^ was measured by nitrocellulose filtration. The peptidyl transferase activity of ribosomes was measured by kinetic analysis of the reaction between complex C and puromycin. The reaction was performed at 25 °C under optimal conditions [20 mM Hepes-KOH, pH 7.5, containing 2.5 mM (CH_3_COO)_2_Mg, 150 mM CH_3_COOK, 3.5 mM spermidine, 6 mM *β*-mercaptoethanol] in the presence of various concentrations of puromycin (Figure S5).

## 5. Conclusions

Our study provides a comprehensive outline of the molecular events that are modulated by known (miRNAs) or new (tRFs) regulators and affect tumor-suppressor and tumor-promoter gene expression. Moreover, it highlights the important regulatory role of tRNAs in the direct or indirect regulation of gene expression and their contribution through the production of tRFs to aberrant signaling and translation deregulation in lung cancer biopsy specimens. Finally, it consolidates the notion that differential expression and simultaneous action of several important regulatory RNAs affects synergistically central cellular processes, like translation.

## Supplementary Materials

The following are available online at https://www.mdpi.com/2072-6694/12/10/3056/s1, Figure S1: Validation of the expression levels of several miRNAs by RT-qPCR, Figure S2: Validation of the expression levels of selected tRFs by RT-Qpcr, Figure S3: Expression level alterations of important RNA related genes, Figure S4: Explanatory illustration of the experimental procedure of the 43S pre-initiation complex formation assays indicating each examined condition, Figure S5: Translation-elongation assays in cell free systems derived from normal and lung cancer tissues, Figure S6: A schematic representation of events that link the expression patterns of miRNAs, tRFs and tRNAs with aberrant signaling and deregulated translation initiation, in lung cancer biopsy specimens, Figure S7: Western blot analysis of mTOR. Uncropped image used for the main Figure 4, Figure S8: Western blot analysis of p-mTOR (Ser2448). Uncropped image used for the main Figure 4, Figure S9: Western blot analysis of p-ERK1/2 (Thr202, Tyr204). Uncropped image used for the main Figure 4, Figure S10: Western blot analysis of *β*-actin (Thr202, Tyr204). Uncropped image used for the main Figure 4, Figure S11: Western blot analysis using anti-puromycin to assess the levels of puromycylated nascent peptides. Uncropped image used for the main Figure 4, Figure S12: Western blot analysis of *β*-actin. Uncropped image used for the main Figure 4, Figure S13: Western blot analysis of P-p38 MAPK (Thr180, Tyr182). Uncropped image used for the main Figure 5, Figure S14: Western blot analysis of P-ERK1/2 (Thr202, Tyr204). Uncropped image used for the main Figure 5, Figure S15: Western blot analysis of P-AKT (Ser473). Uncropped image used for the main Figure 5, Figure S16: Western blot analysis of mTOR. Uncropped image used for the main Figure 5, Figure S17: Western blot analysis of P-mTOR (Ser2448). Uncropped image used for the main Figure 5, Figure S18: Western blot analysis of P-p70 S6 kinase (Thr389). Uncropped image used for the main Figure 5, Figure S19: Western blot analysis of P-S6 (Ser235/236). Uncropped image used for the main Figure 5, Figure S20: Western blot analysis of c-MYC. Uncropped image used for the main Figure 5, Figure S21: Western blot analysis of *β*-actin. Uncropped image used for the main Figure 5, Figure S22: Western blot analysis of 4EBP1. Uncropped image used for the main Figure 5, Figure S23: Western blot analysis of P-4EBP1 (Thr37/46). Uncropped image used for the main Figure 5, Figure S24: Western blot analysis of P-4EBP1 (Thr70). Uncropped image used for the main Figure 5, Figure S25: Western blot analysis of P-4EBP1 (Ser65). Uncropped image used for the main Figure 5, Figure S26: Western blot analysis of eIF4E. Uncropped image used for the main Figure 5, Figure S27: Western blot analysis of P-eIF4E (Ser209). Uncropped image used for the main Figure 5, Figure S28: Western blot analysis of eIF2α. Uncropped image used for the main Figure 5, Figure S29: Western blot analysis of P-eIF2α (Ser51). Uncropped image used for the main Figure 5, Figure S30: Western blot analysis of eEF1A. Uncropped image used for the main Figure 5, Figure S31: Western blot analysis of eEF2. Uncropped image used for the main Figure 5, Figure S32: Western blot analysis of *β*-actin. Uncropped image used for the main Figure 5, Figure S33: Polysome profiling Normal fractions (2% agarose gel). RNA extracted from fractions 1-13, Figure S34: Polysome profiling Tumor fractions (2% agarose gel). RNA extracted from fractions 1–13. Table S1: List of most significantly altered miRNAs in NSCLC is provided as a separate file (.xlsx format), Table S2: List of most significantly altered tRFs in NSCLC is provided as a separate file (.xlsx format), Table S3: List of identified tRFs along with their predicted targets is provided as a separate file (.xlsx format), Table S4: List of most significantly altered tRNAs in NSCLC is provided as a separate file (.xlsx format), Table S5: List of most significantly affected genes after transfection of tRF-5003b in A549 cells is provided as a separate file (.xlsx format), Table S6: List of most significantly affected genes after transfection of tRF-3021a in A549 cells is provided as a separate file (.xlsx format), Table S7: Baseline characteristics of lung adenocarcinoma patients, Table S8: List of primers used for miRNAs expression analysis, Table S9: List of primers used for tRFs expression analysis, Table S10: List of primers used for gene expression analysis, Table S11: List of antibodies used for Western blot analysis, Table S12: List of siRNAs for overexpression experiments.
