# Supplementary material for: Contribution of miRNAs, tRNAs and tRFs to Aberrant Signaling and Translation Deregulation in Lung Cancer
Source: Cancers (Basel). 2020 Oct 20;12(10):3056. doi: 10.3390/cancers12103056 (PMC7593945; doi:10.3390/cancers12103056)
Supplement: Supplementary file 1 [file cancers-12-03056-s001.zip › cancers-933468-supple-XML/cancers-933468-supple-XML.pdf]

Supplementary Materials: Contribution of miRNAs, tRNAs and tRFs to Aberrant Signaling and Translation Deregulation in Lung Cancer

Ilias Skeparnias, Dimitrios Anastasakis, Katerina Grafanaki, George Kyriakopoulos, Panagiotis Alexopoulo, Dimitrios Dougenis, Andreas Scorilas, Christos Kontos and Constantinos Stathopoulos

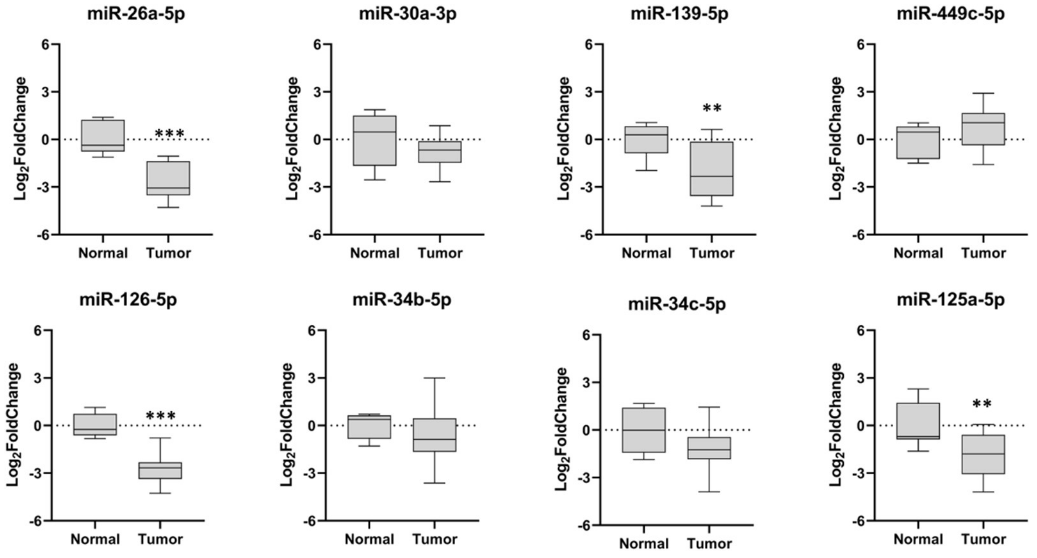

Figure S1. Validation of the expression levels of several miRNAs by RT-qPCR. A total of 8 samples were analysed (3 normal and 5 tumor tissue specimens) for validation of 8 miRNAs after the NGS analysis. Normalization and subsequent calculation of log2FoldChange were performed using miR-103 as calibrators and −ΔΔCt method. All experiments were performed in triplicates. Asterisks represent *p*-values after unpaired t test between the log2fold expression of each miRNA in normal and tumor specimens (* $p < 0.05$; ** $p < 0.01$; *** $p < 0.001$).

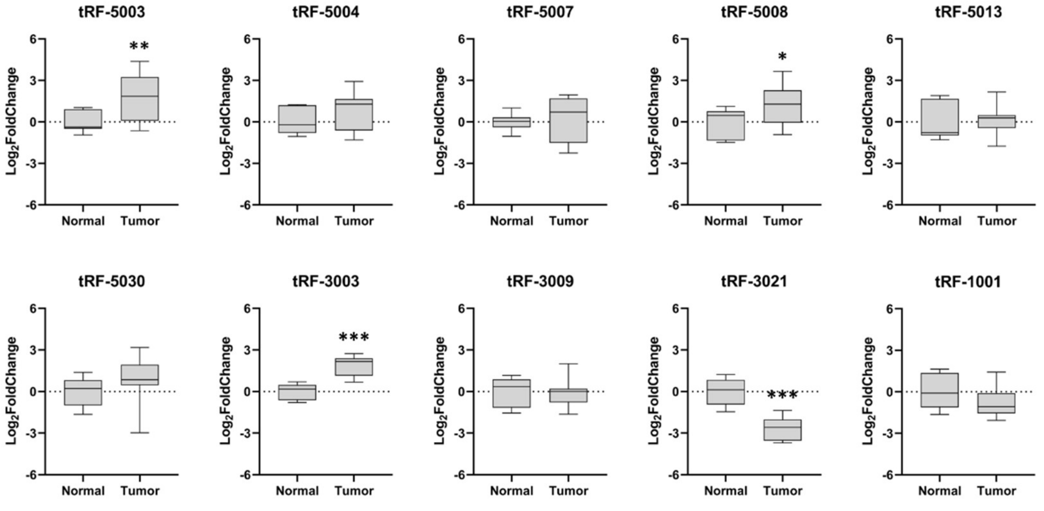

Figure S2. Validation of the expression levels of selected tRFs by RT-qPCR. A total of 8 samples were analyzed (3 normal and 5 tumor tissue specimens) for validation of 10 tRFs after the NGS analysis. Normalization and subsequent calculation of log2FoldChange were performed using miR-103 as calibrators and −ΔΔCt method. All experiments were performed in triplicates. Asterisks represent *p*-

values after unpaired t test between the log2fold expression of each tRF in normal and tumor specimens (* $p < 0.05$; ** $p < 0.01$; *** $p < 0.001$).

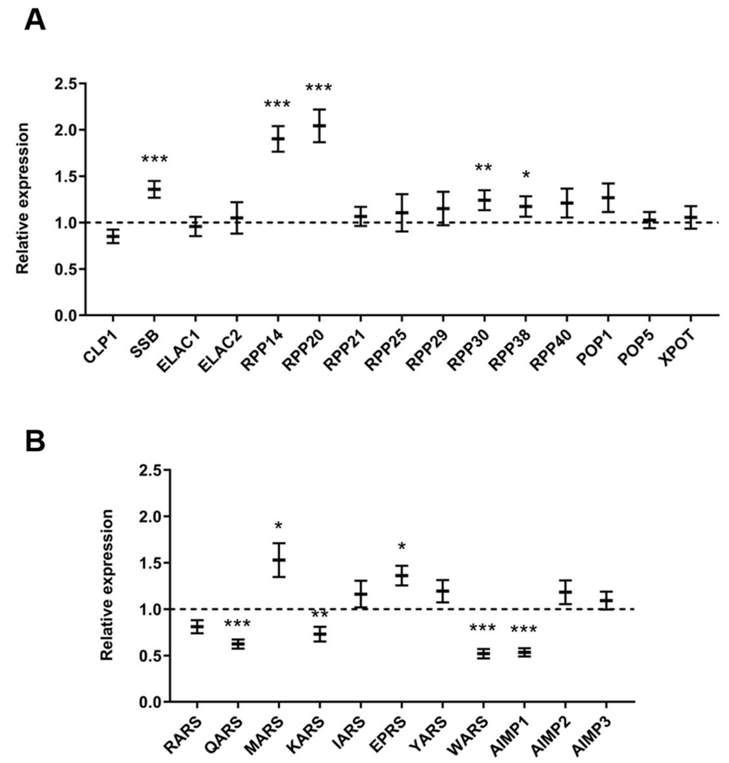

Figure S3. Expression level alterations of important RNA related genes. (**A**) tRNA biogenesis related genes were examined for their relative expression in lung cancer. *SSB*, *RPP14*, *RPP20*, *RPP30* and *RPP38* were found significantly upregulated in lung cancer biopsies. (**B**) mRNA expression levels of genes responsible for aminoacylation of tRNA molecules were also analyzed. *MARS* and *EPRS* expression were found elevated, whereas we observed downregulation of *WARS*, *QARS*, *KARS* and *AIMP1* expression in tumor specimens. All experiments were performed in triplicates, bar graphs represent mean ± SEM (error bars) and one-way Anova was used for the statistical analysis (* $p < 0.05$; ** $p < 0.01$; *** $p < 0.001$).

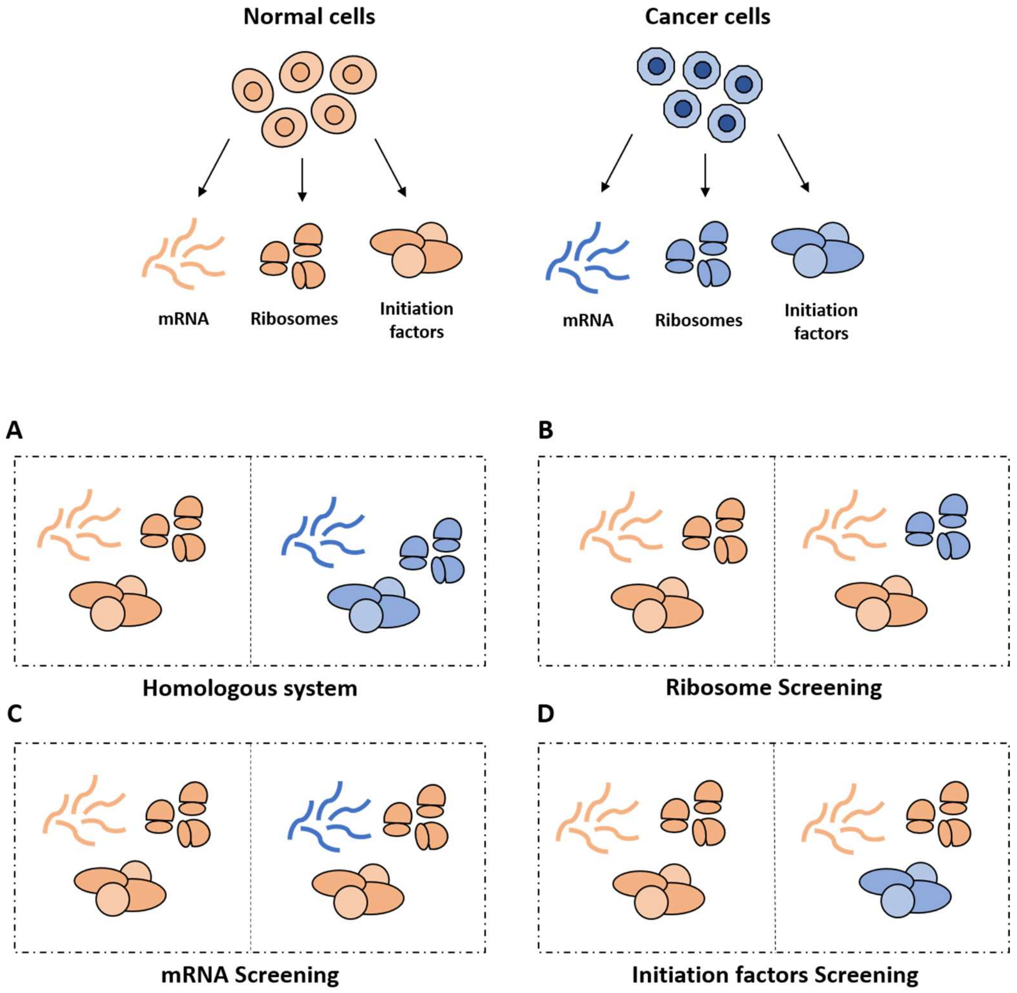

Figure S4. Explanatory illustration of the experimental procedure of the 43S pre-initiation complex formation assays indicating each examined condition. (**A** to **D**) the origin of the components which derived either from normal (brown color) or tumor specimens (blue color).

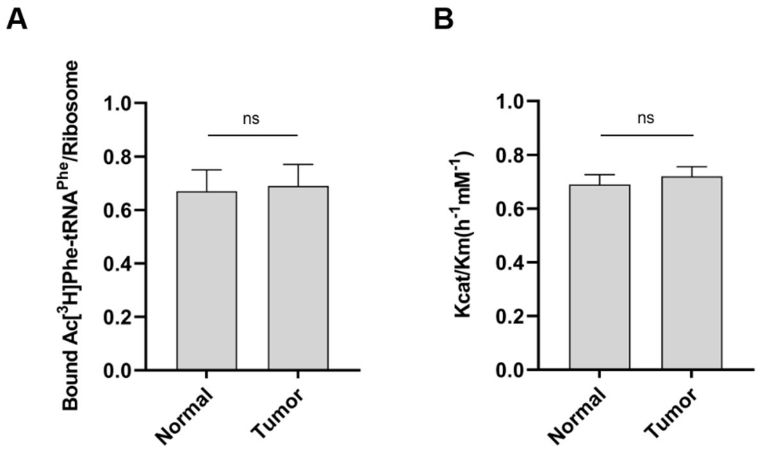

Figure S5. Translation-elongation assays in cell free systems derived from normal and lung cancer tissues. (**A**) Binding of Ac[^{3}H]Phe-tRNA^{Phe} to the A-site of poly(U)-programmed 80S ribosomes prefilled with tRNA^{Phe} at the *p*-site. (**B**) Catalytic activity of peptidyl transferase estimated from kinetic data that were collected from a reaction between complex C and excess of puromycin. Unpaired t test

between the relative values between normal and tumor specimens showed no statistically significant alterations (ns, not significant).

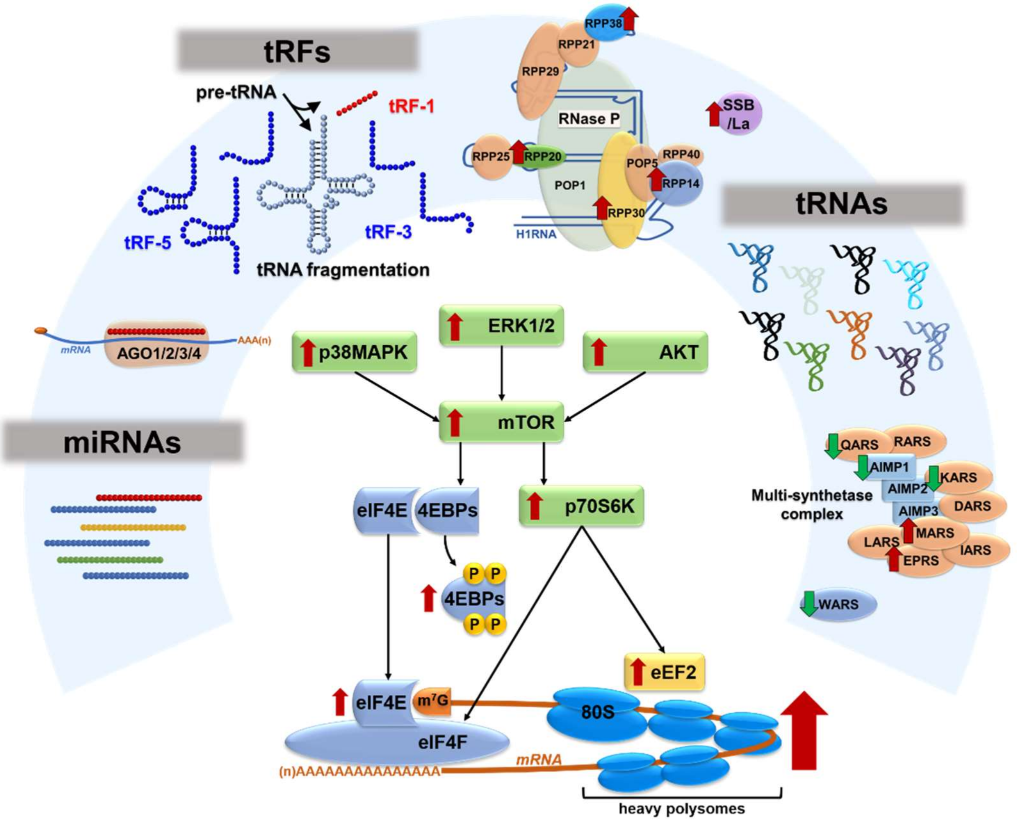

Figure S6. A schematic representation of events that link the expression patterns of miRNAs, tRFs and tRNAs with aberrant signaling and deregulated translation initiation, in lung cancer biopsy specimens.

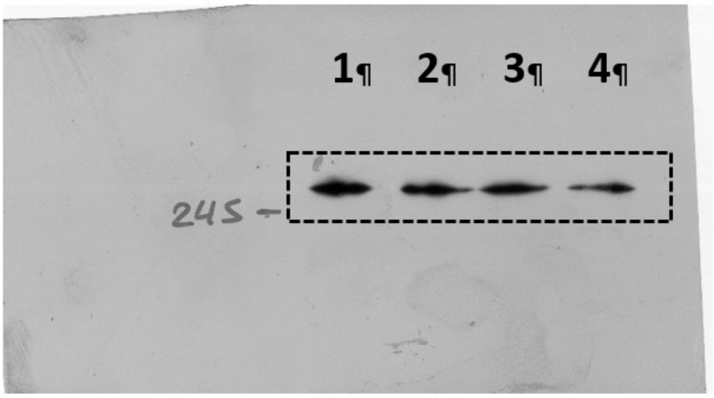

Figure S7. Western blot analysis of mTOR. Uncropped image used for the main Figure 4. Samples order: 1: si Luciferase; 2: tRF-5003b; 3: si Luciferase; 4: tRF-3021a.

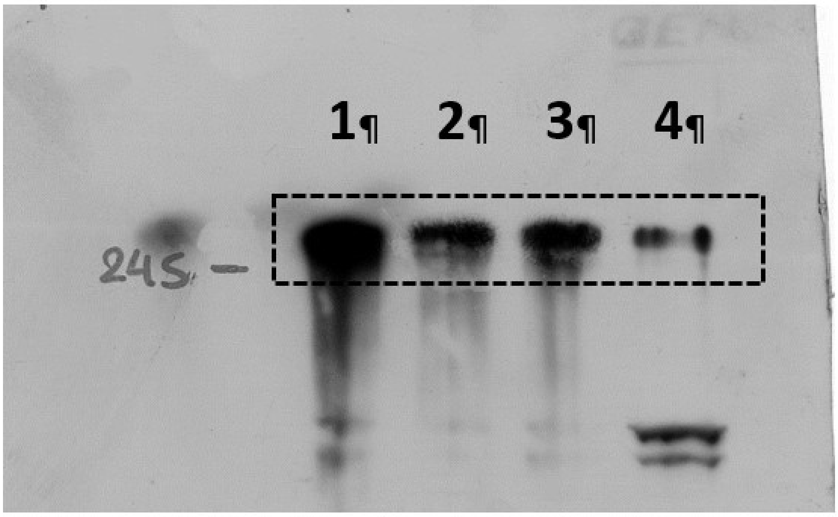

Figure S8. Western blot analysis of p-mTOR (Ser2448). Uncropped image used for the main Figure 4. Samples order: 1: si Luciferase; 2: tRF-5003b; 3: si Luciferase; 4: tRF-3021a.

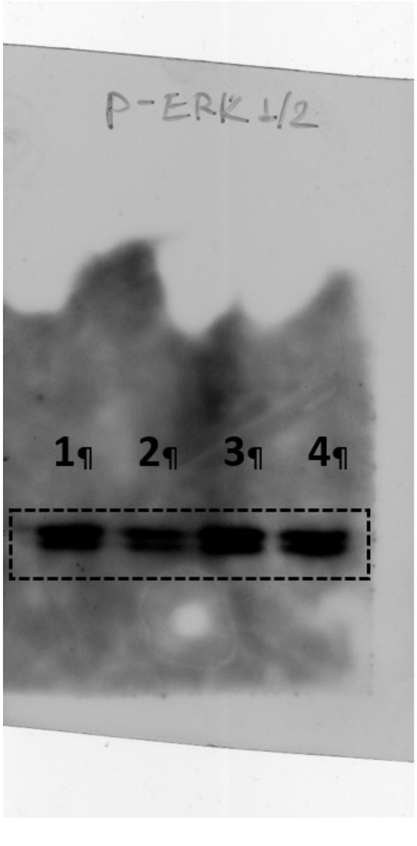

Figure S9. Western blot analysis of p-ERK1/2 (Thr202, Tyr204). Uncropped image used for the main Figure 4. Samples order: 1: si Luciferase; 2: tRF-5003b; 3: si Luciferase; 4: tRF-3021a.

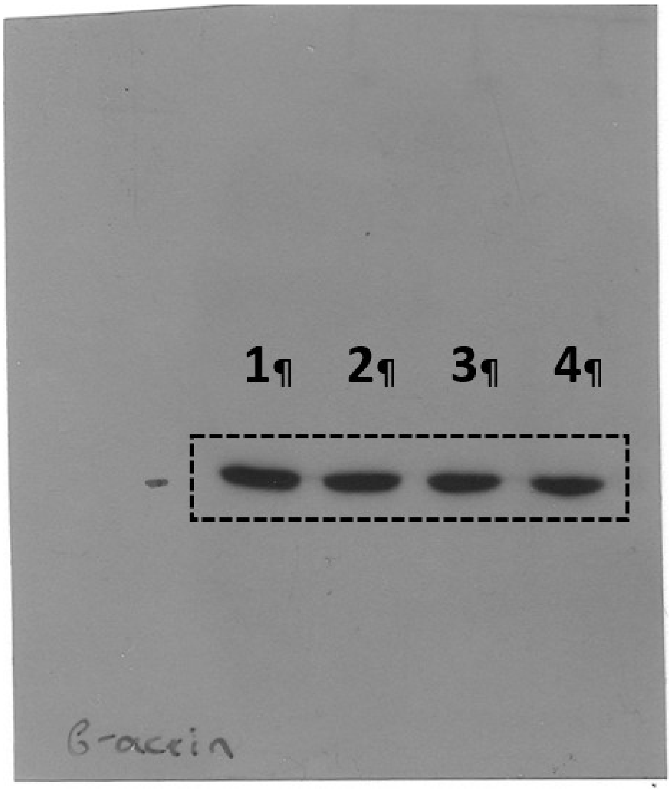

Figure S10. Western blot analysis of β-actin (Thr202, Tyr204). Uncropped image used for the main Figure 4. Samples order: 1: si Luciferase; 2: tRF-5003b; 3: si Luciferase; 4: tRF-3021a.

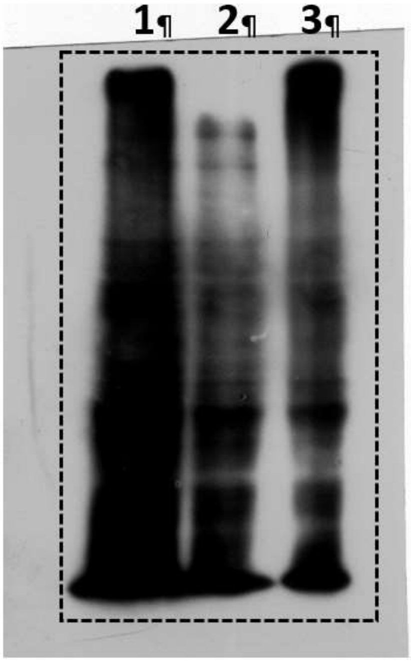

Figure S11. Western blot analysis using anti-puromycin to assess the levels of puromycylated nascent peptides. Uncropped image used for the main Figure 4. Samples order: 1: si Luciferase; 2: tRF-5003b; 3: tRF-3021a.

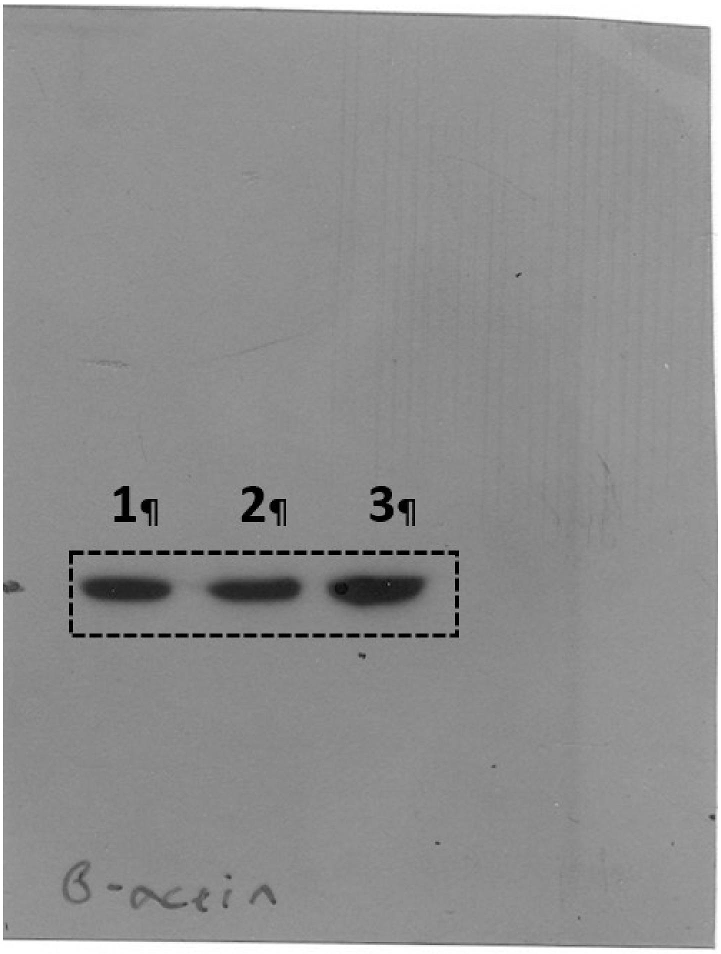

Figure S12. Western blot analysis of *β*-actin. Uncropped image used for the main Figure 4. Samples order: 1: si Luciferase; 2: tRF-5003b; 3: tRF-3021a.

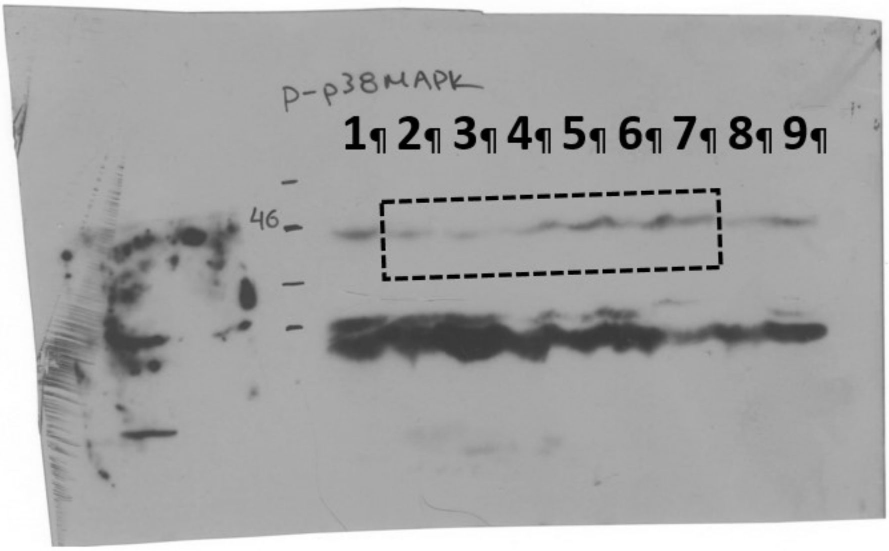

Figure S13. Western blot analysis of P-p38 MAPK (Thr180, Tyr182). Uncropped image used for the main Figure 5. Samples order: 1: A549 cell extract (positive control); 2: Normal 1; 3: Normal 2; 4: Normal 3; 5: Tumor 1; 6: Tumor 2; 7: Tumor 3; 8: Tumor 4; 9: CL1-5 cell extract (positive control).

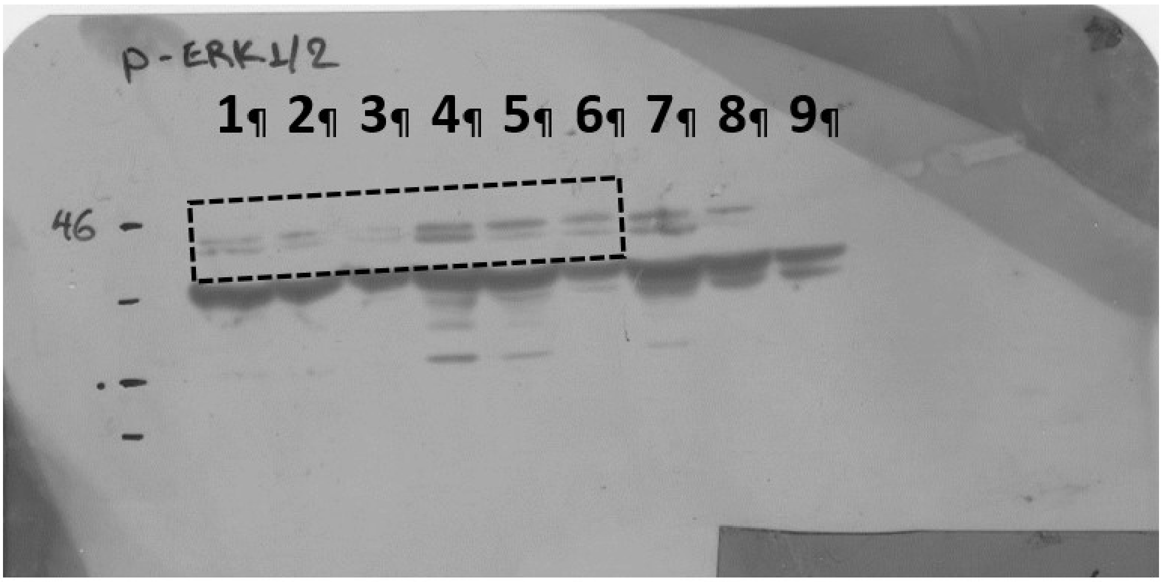

Figure S14. Western blot analysis of P-ERK1/2 (Thr202, Tyr204). Uncropped image used for the main Figure 5. Samples order: 1: Normal 1; 2: Normal 2; 3: Normal 3; 4: Tumor 1; 5: Tumor 2; 6: Tumor 3; 7: Tumor 4; 8: A549 cell extract (positive control); 9: CL1-5 cell extract (positive control).

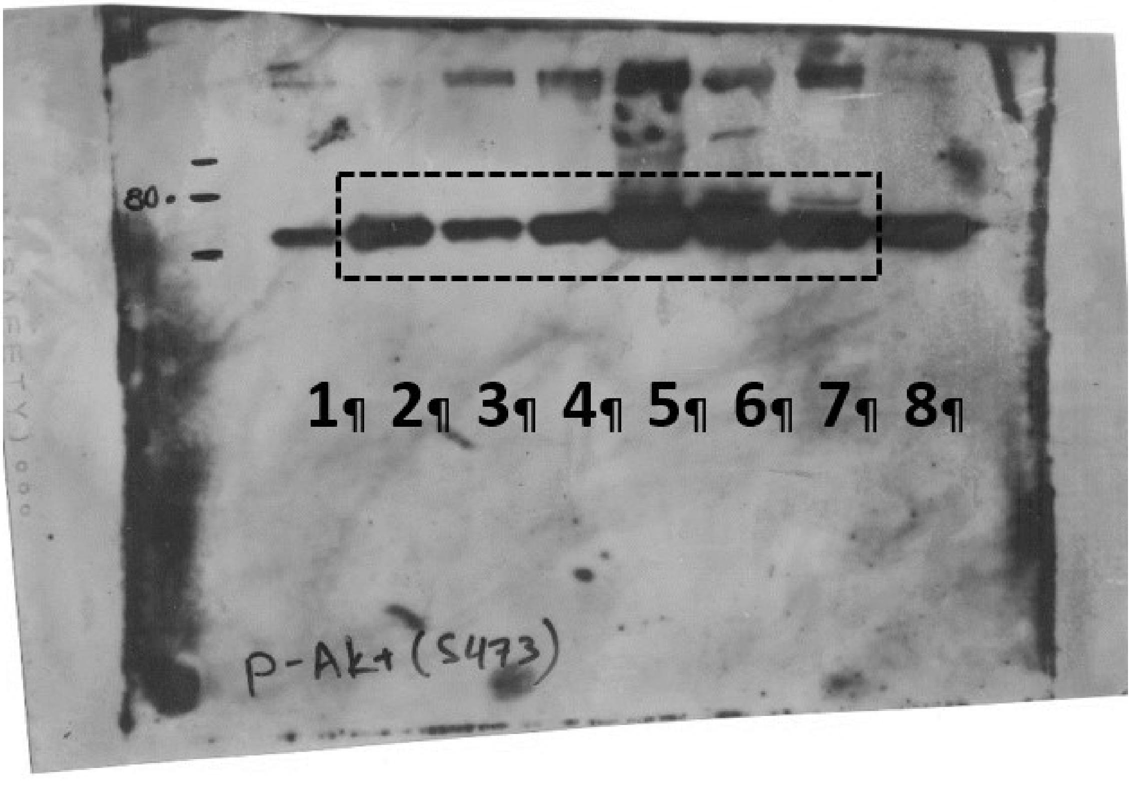

Figure S15. Western blot analysis of P-AKT (Ser473). Uncropped image used for the main Figure 5. Samples order: 1: A549 cell extract (positive control); 2: Normal 1; 3: Normal 2; 4: Normal 3; 5: Tumor 1; 6: Tumor 2; 7: Tumor 3; 8: Tumor 4.

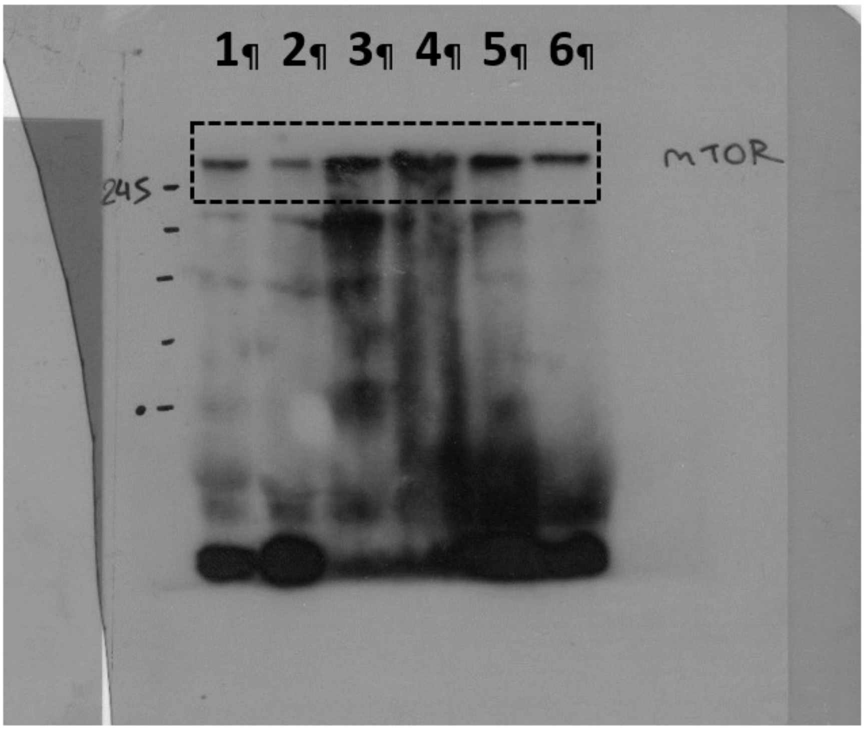

Figure S16. Western blot analysis of mTOR. Uncropped image used for the main Figure 5. Samples order: 1: Normal 1; 2: Normal 2; 3: Normal 3; 4: Tumor 1; 5: Tumor 2; 6: Tumor 3.

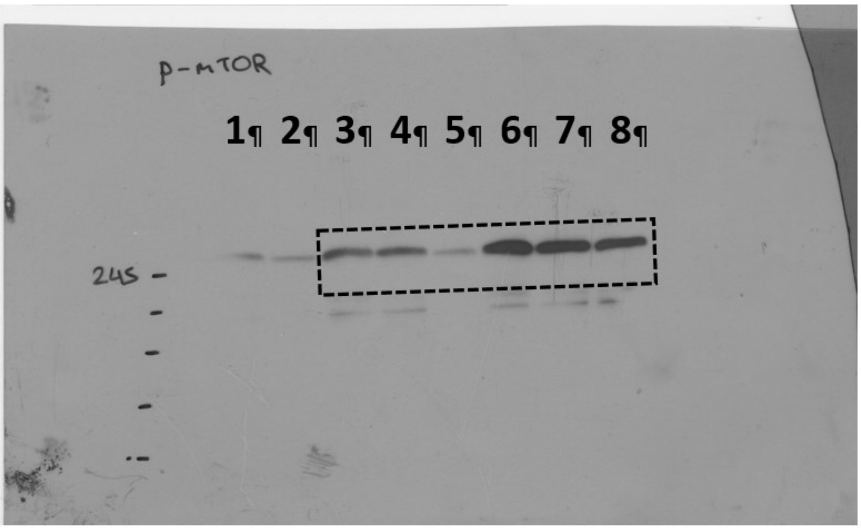

Figure S17. Western blot analysis of P-mTOR (Ser2448). Uncropped image used for the main Figure 5. Samples order: 1: A549 cell extract (positive control); 2: CL1-5 cell extract (positive control); 3: Normal 1; 4: Normal 2; 5: Normal 3; 6: Tumor 1; 7: Tumor 2; 8: Tumor 3.

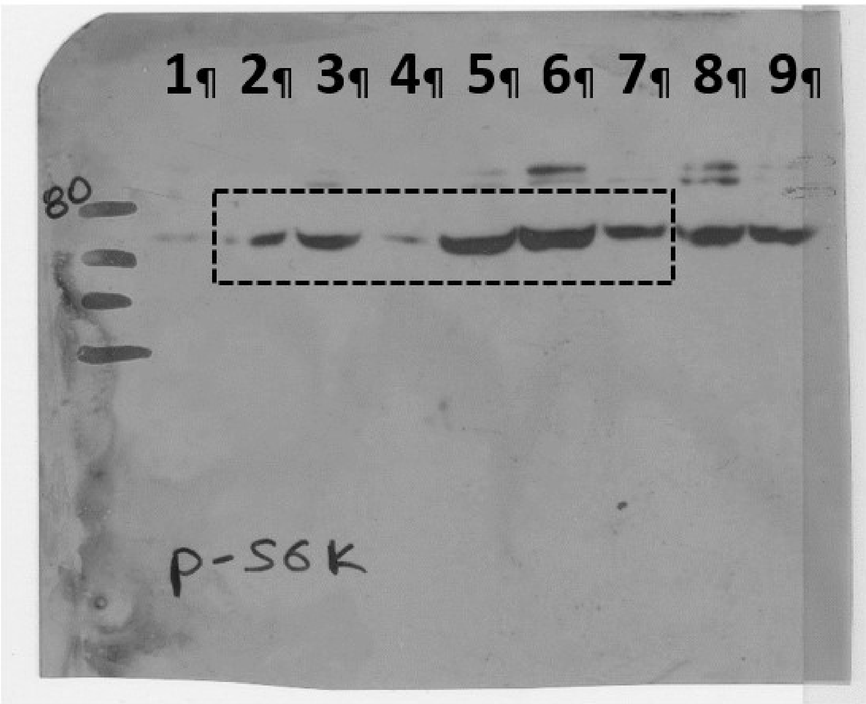

Figure S18. Western blot analysis of P-p70 S6 kinase (Thr389). Uncropped image used for the main Figure 5. Samples order: 1: A549 cell extract (positive control); 2: Normal 1; 3: Normal 2; 4: Normal 3; 5: Tumor 1; 6: Tumor 2; 7: Tumor 3; 8: Tumor 4; 9: CL1-5 cell extract (positive control).

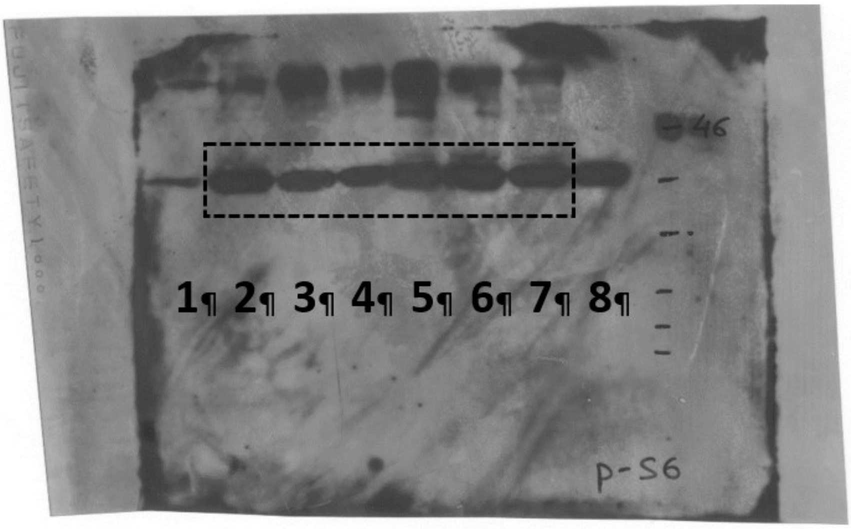

Figure S19. Western blot analysis of P-S6 (Ser235/236). Uncropped image used for the main Figure 5. Samples order: 1: A549 cell extract (positive control); 2: Normal 1; 3: Normal 2; 4: Normal 3; 5: Tumor 1; 6: Tumor 2; 7: Tumor 3; 8: Tumor 4.

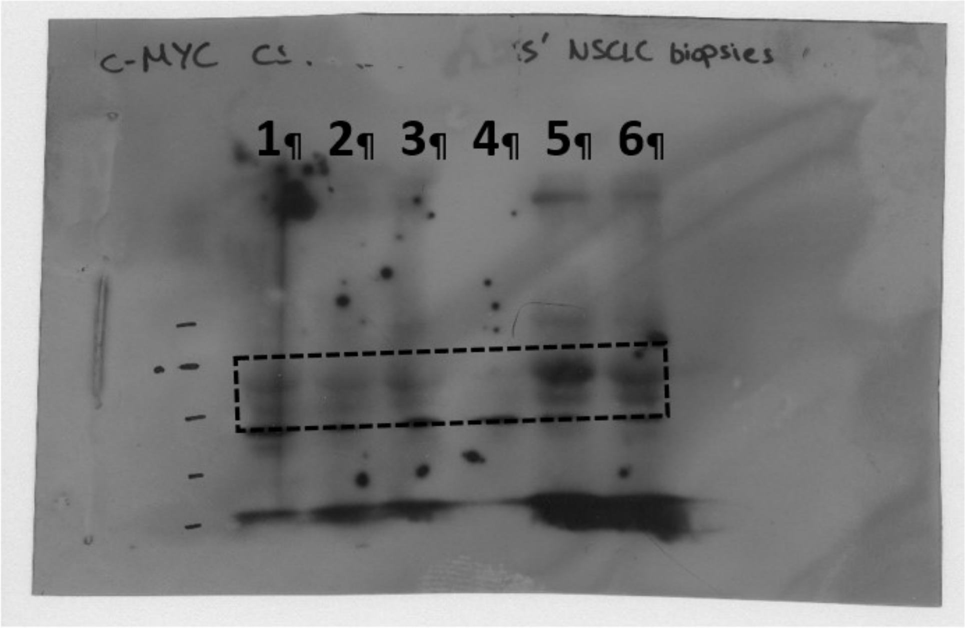

Figure S20. Western blot analysis of c-MYC. Uncropped image used for the main Figure 5. Samples order: 1: Normal 1; 2: Normal 2; 3: Normal 3; 4: Tumor 1; 5: Tumor 2; 6: Tumor 3.

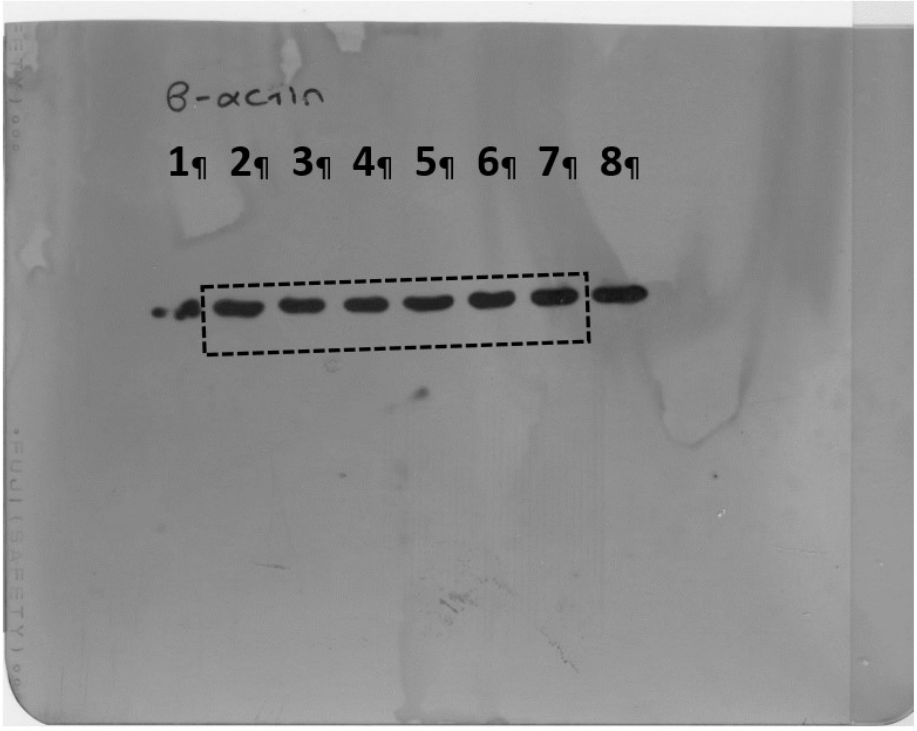

Figure S21. Western blot analysis of *β*-actin. Uncropped image used for the main Figure 5. Samples order: 1: A549 cell extract (positive control); 2: Normal 1; 3: Normal 2; 4: Normal 3; 5: Tumor 1; 6: Tumor 2; 7: Tumor 3; 8: Tumor 4.

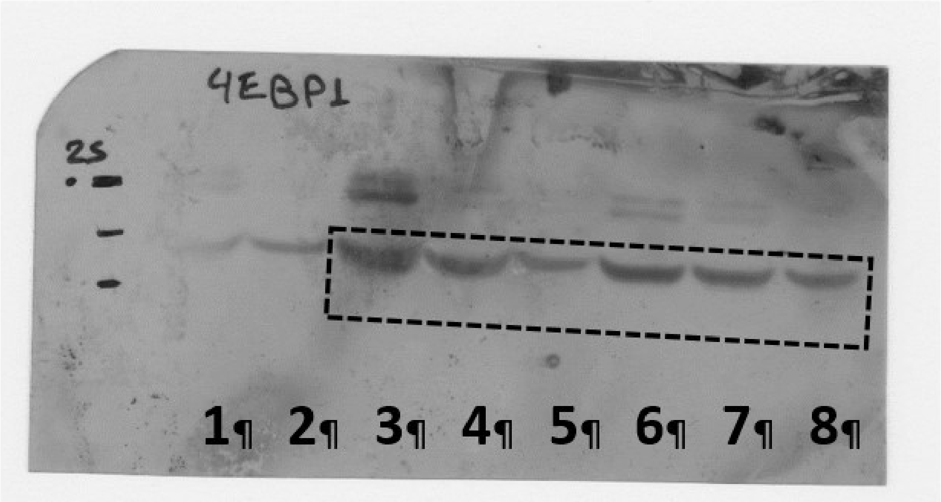

Figure S22. Western blot analysis of 4EBP1. Uncropped image used for the main Figure 5. Samples order: 1: A549 cell extract (positive control); 2: CL1-5 cell extract (positive control); 3: Normal 1; 4: Normal 2; 5: Normal 3; 6: Tumor 1; 7: Tumor 2; 8: Tumor 3.

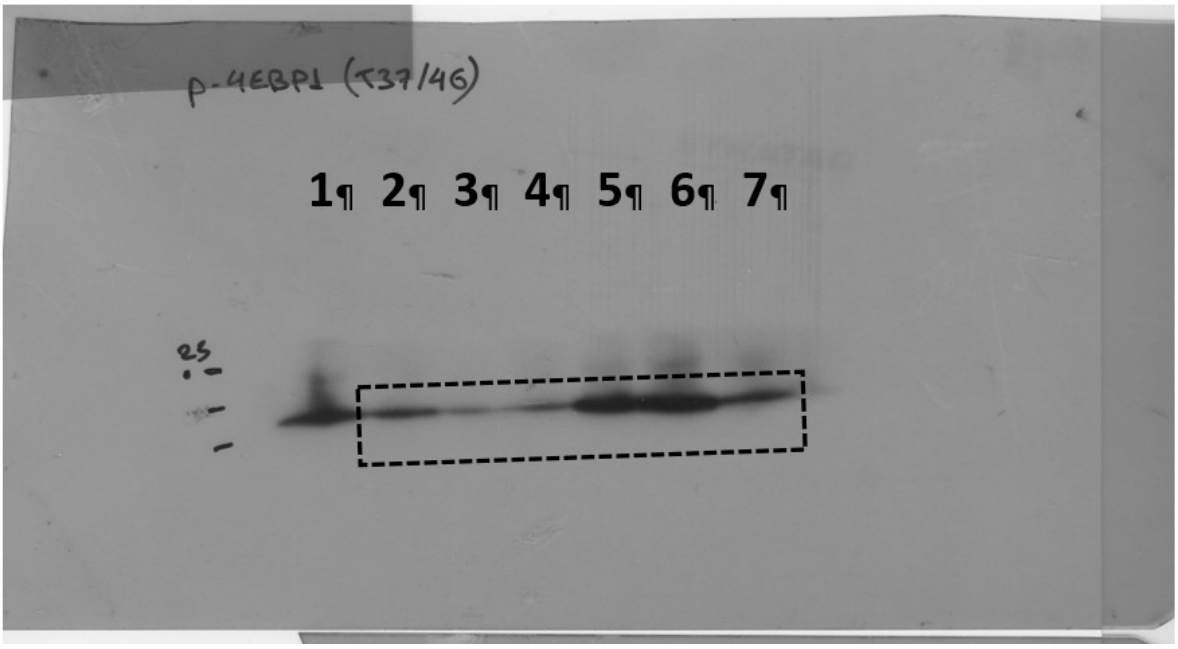

Figure S23. Western blot analysis of P-4EBP1 (Thr37/46). Uncropped image used for the main Figure 5. Samples order: 1: A549 cell extract (positive control); 2: Normal 1; 3: Normal 2; 4: Normal 3; 5: Tumor 1; 6: Tumor 2; 7: Tumor 3.

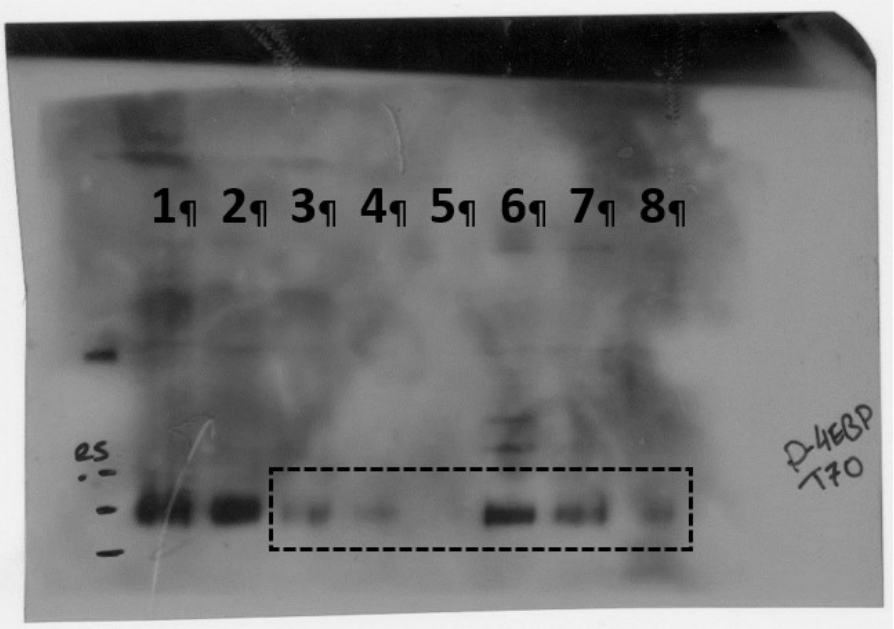

Figure S24. Western blot analysis of P-4EBP1 (Thr70). Uncropped image used for the main Figure 5. Samples order: 1: A549 cell extract (positive control); 2: CL1-5 cell extract (positive control); 3: Normal 1; 4: Normal 2; 5: Normal 3; 6: Tumor 1; 7: Tumor 2; 8: Tumor 3.

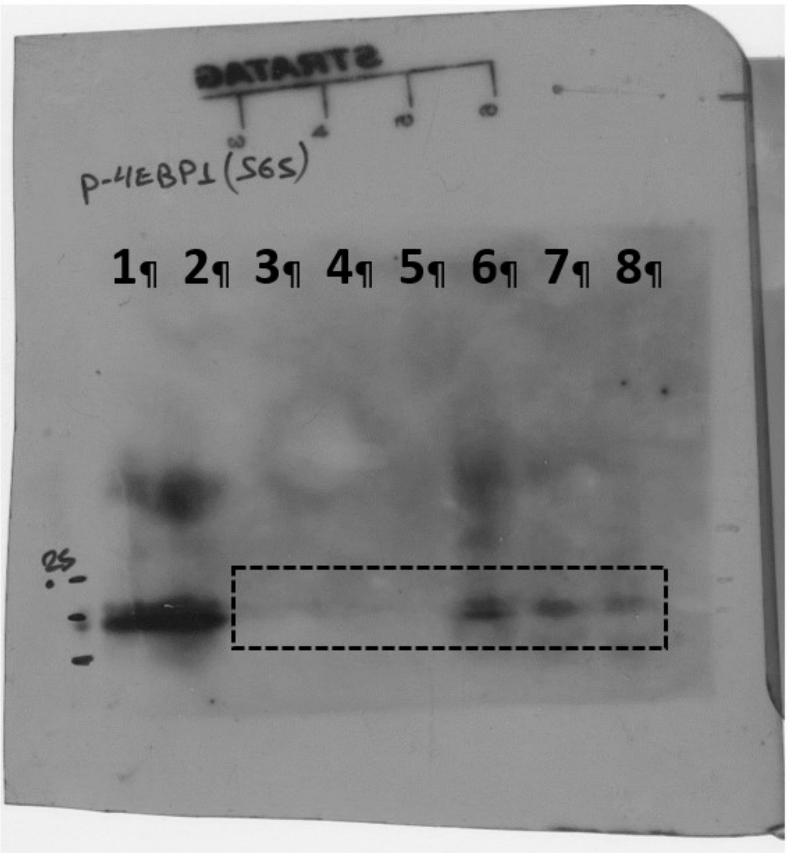

Figure S25. Western blot analysis of P-4EBP1 (Ser65). Uncropped image used for the main Figure 5. Samples order: 1: A549 cell extract (positive control); 2: CL1-5 cell extract (positive control); 3: Normal 1; 4: Normal 2; 5: Normal 3; 6: Tumor 1; 7: Tumor 2; 8: Tumor 3.

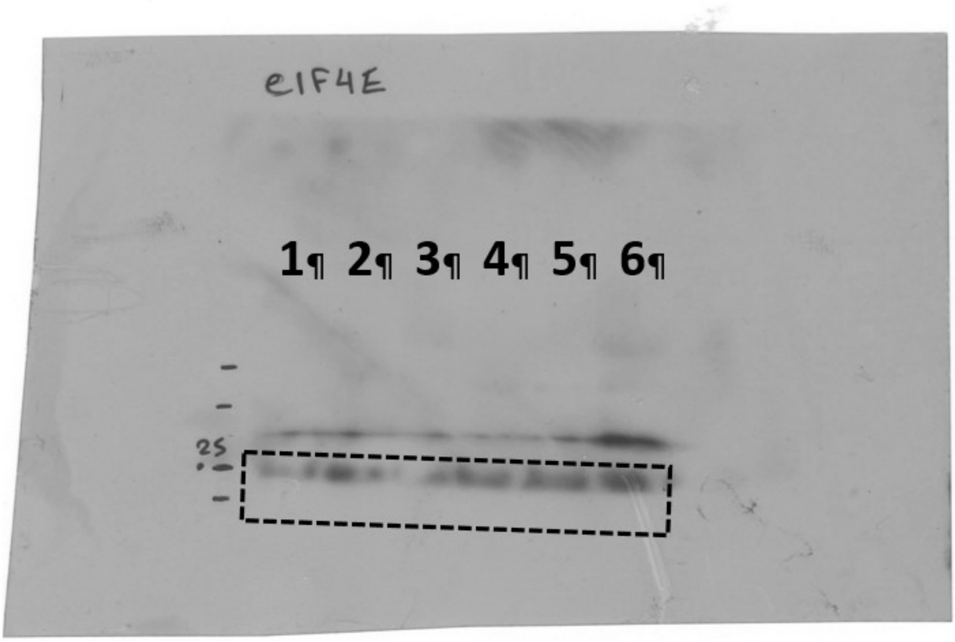

Figure S26. Western blot analysis of eIF4E. Uncropped image used for the main Figure 5. Samples order: 1: Normal 1; 2: Normal 2l; 3: Normal 3; 4: Tumor 1; 5: Tumor 2; 6: Tumor 3.

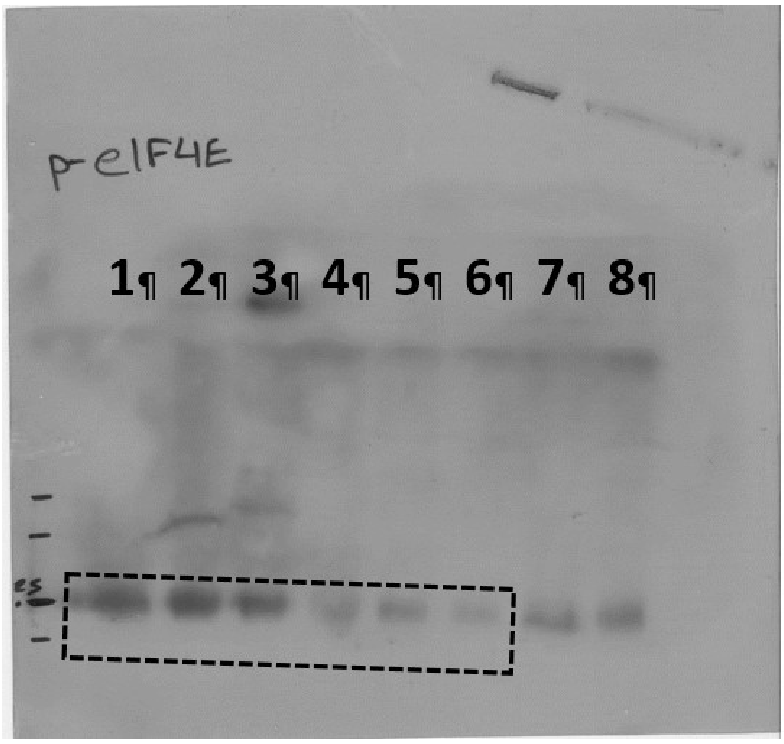

Figure S27. Western blot analysis of P-eIF4E (Ser209). Uncropped image used for the main Figure 5. Samples order: 1: Normal 1; 2: Normal 2l; 3: Normal 3; 4: Tumor 1; 5: Tumor 2; 6: Tumor 3; 7: A549 cell extract (positive control); 8: CL1-5 cell extract (positive control).

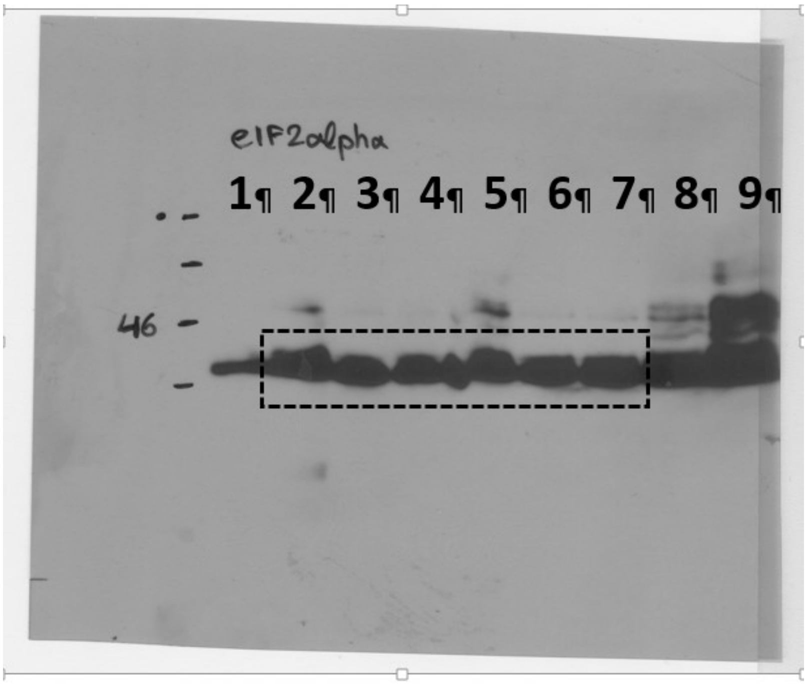

Figure S28. Western blot analysis of eIF2α. Uncropped image used for the main Figure 5. Samples order: 1: A549 cell extract (positive control); 2: Normal 1; 3: Normal 2; 4: Normal 3; 5: Tumor 1; 6: Tumor 2; 7: Tumor 3; 8: Tumor 4; 9: CL1-5 cell extract (positive control).

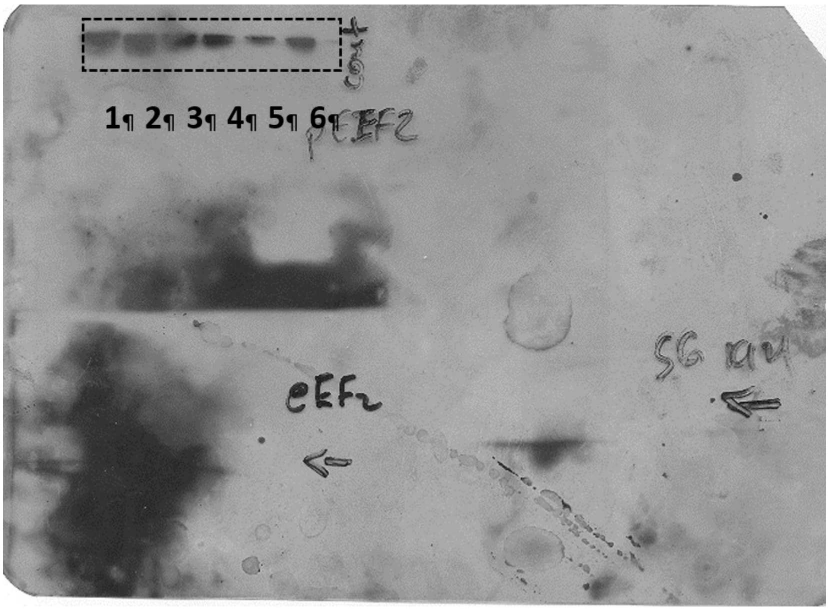

Figure S29. Western blot analysis of P-eIF2α (Ser51). Uncropped image used for the main Figure 5. Samples order: 1: Normal 1; 2: Normal 2; 3: Normal 3; 4: Tumor 1; 5: Tumor 2; 6: Tumor 3.

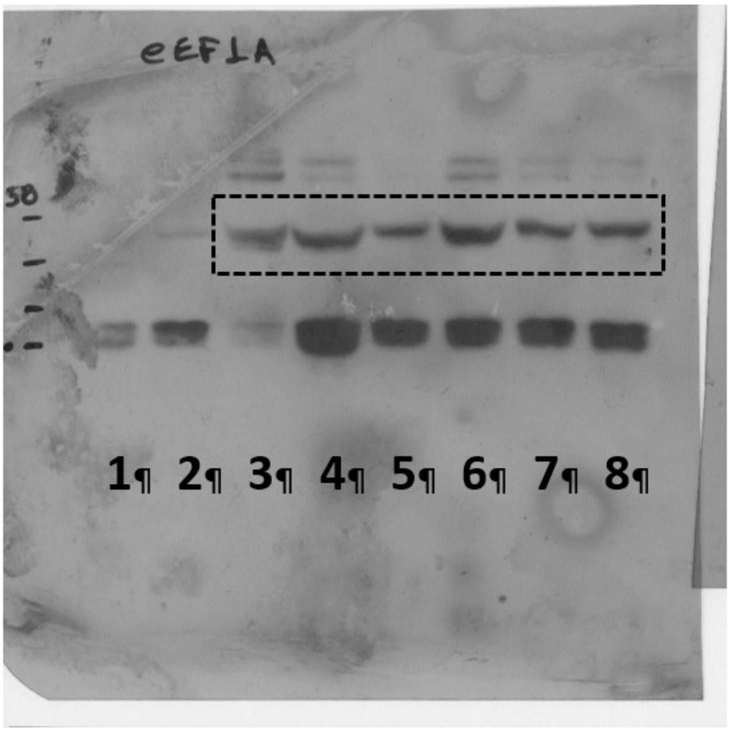

Figure S30. Western blot analysis of eEF1A. Uncropped image used for the main Figure 5. Samples order: 1: A549 cell extract (positive control); 2: CL1-5 cell extract (positive control); 3: Normal 1; 4: Normal 2; 5: Normal 3; 6: Tumor 1; 7: Tumor 2; 8: Tumor 3.

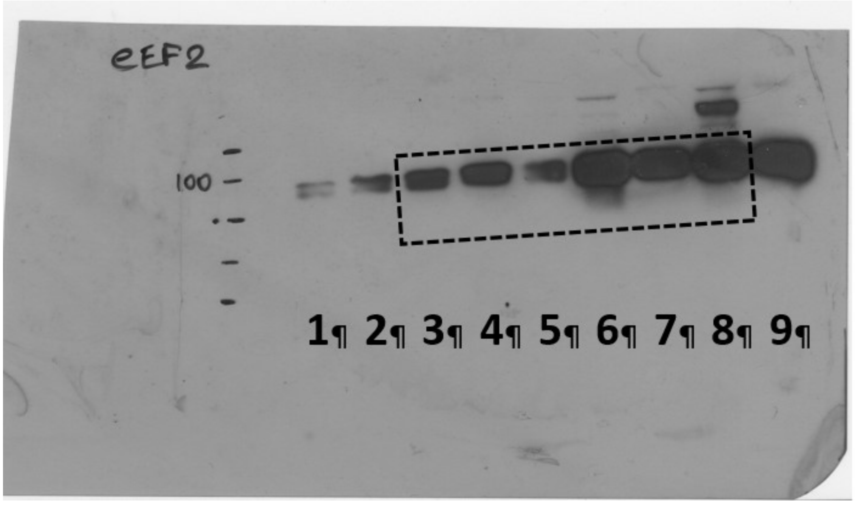

Figure S31. Western blot analysis of eEF2. Uncropped image used for the main Figure 5. Samples order: 1: A549 cell extract (positive control); 2: CL1-5 cell extract (positive control); 3: Normal 1; 4: Normal 2; 5: Normal 3; 6: Tumor 1; 7: Tumor 2; 8: Tumor 3.

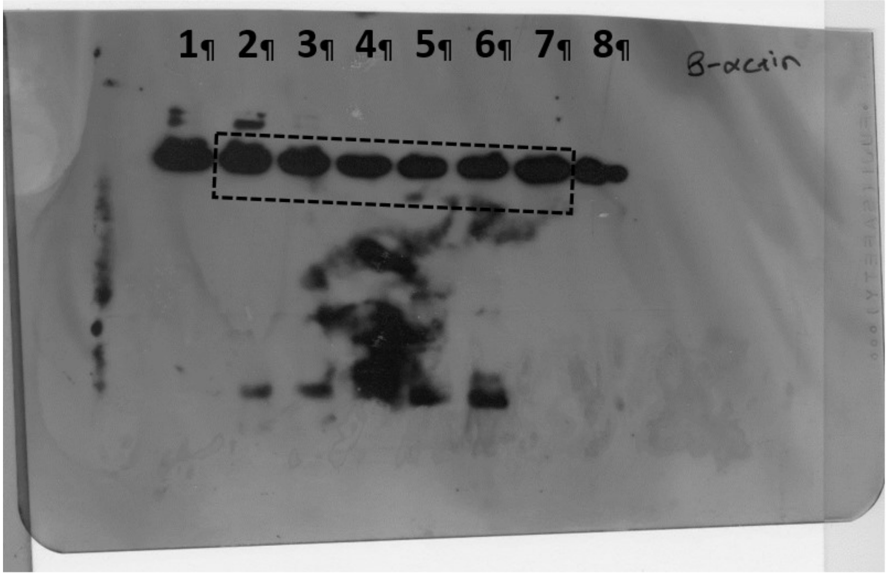

Figure S32. Western blot analysis of *β*-actin. Uncropped image used for the main Figure 5. Samples order: 1: A549 cell extract (positive control); 2: Normal 1; 3: Normal 2; 4: Normal 3; 5: Tumor 1; 6: Tumor 2; 7: Tumor 3; 8: Tumor 4.

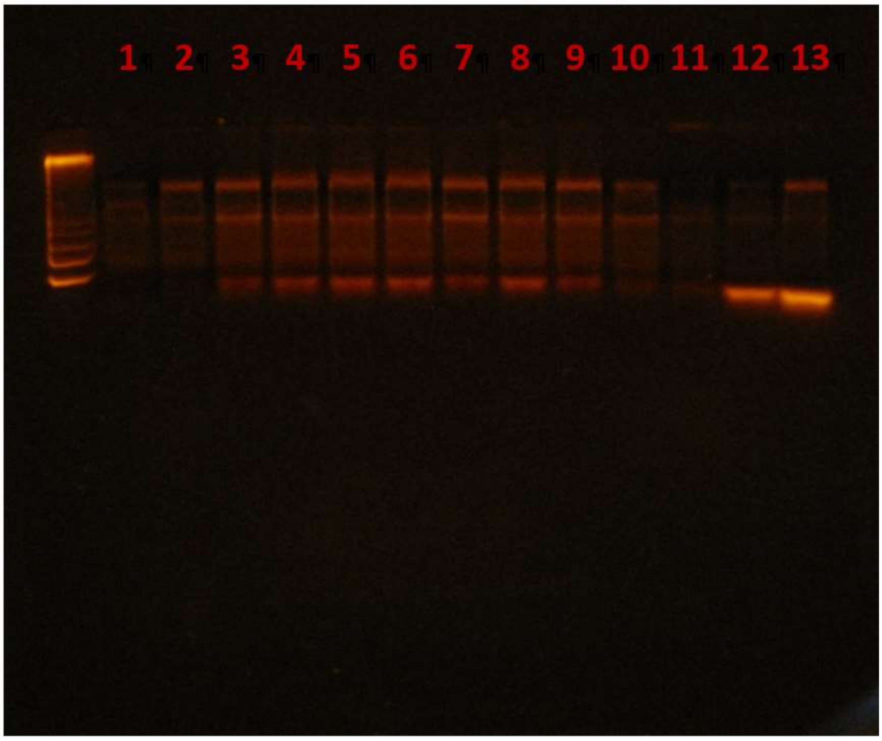

Figure S33. Polysome profiling Normal fractions (2% agarose gel). RNA extracted from fractions 1-13.

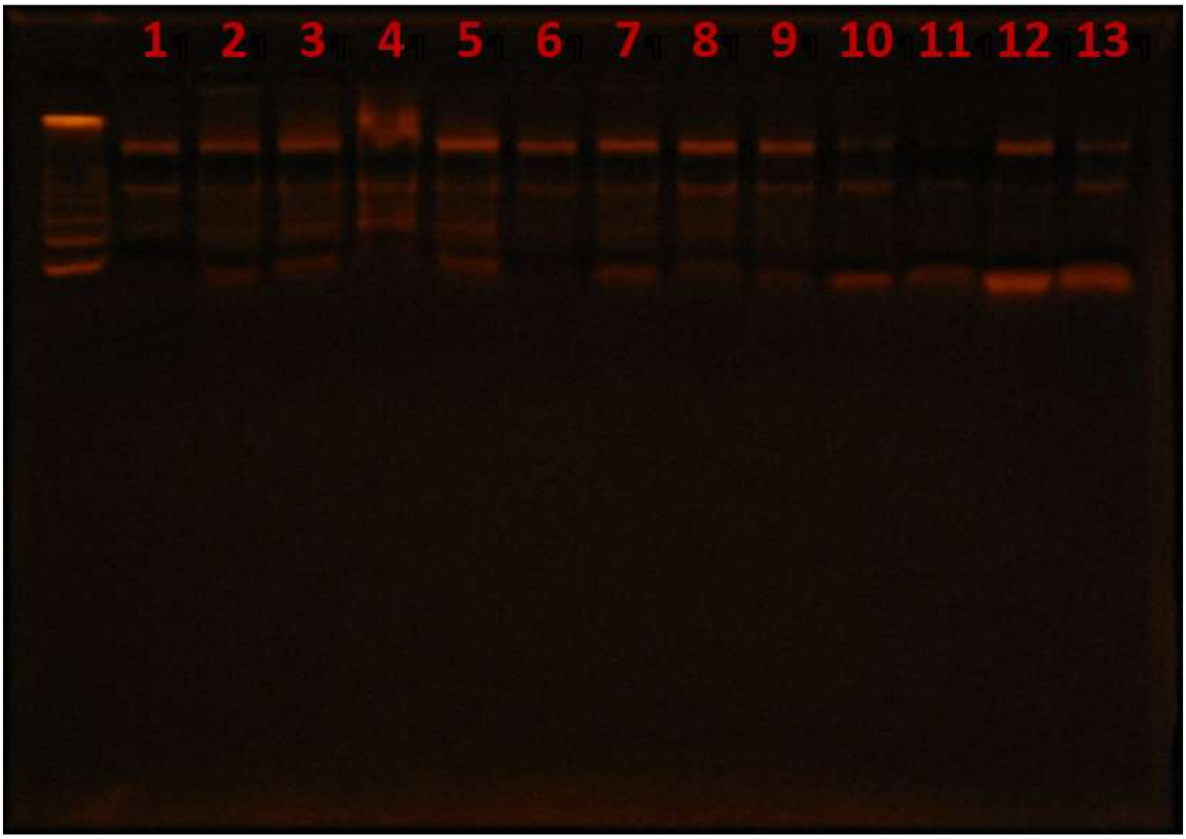

Figure S34. Polysome profiling Tumor fractions (2% agarose gel). RNA extracted from fractions 1–13.

Table S1. List of most significantly altered miRNAs in NSCLC is provided as a separate file (.xlsx format).

Table S2. List of most significantly altered tRFs in NSCLC is provided as a separate file (.xlsx format).

Table S3. List of identified tRFs along with their predicted targets is provided as a separate file (.xlsx format).

Table S4. List of most significantly altered tRNAs in NSCLC is provided as a separate file (.xlsx format).

Table S5. List of most significantly affected genes after transfection of tRF-5003b in A549 cells is provided as a separate file (.xlsx format).

Table S6. List of most significantly affected genes after transfection of tRF-3021a in A549 cells is provided as a separate file (.xlsx format).

Table S7. Baseline characteristics of lung adenocarcinoma patients.

Patient	Gender	Age	Adjacent Normal Tissue	Staging	Smoker
1	F	56	Yes	pT4N2	N/A
2*#	M	45	Yes	pT1bN0	Yes
3*	M	59	Yes	pT2aN1	Former
4*#	F	52	Yes	pT2aN0	Yes
5	F	77	—	pT2aN0	No
6	F	54	Yes	pT3N1	Yes
7	F	77	Yes	pT2N0	Former

8*	F	57	Yes	pT2aN1	Former
9	M	71	—	pT3N2	Former
10	F	76	—	pT1bN1	No
11	M	72	Yes	pT2aN1	No
12	M	68	—	pT2aN0	N/A
13*#	M	48	Yes	pT1bN0	Former
14	M	62	—	pT2aN1	Former
15	M	74	Yes	pT2aN0	Former
16	M	64	Yes	pT4N2	Yes
17	M	69	—	pT2Nx	Yes
18	M	58	Yes	pT2aN0	Yes

* Specimens from patients 2, 3, 4, 8 and 13 were used for the NGS analyses. # Specimens from patients 2, 4 and 13 were used for Western blot analyses and their adjacent normal tissues were used as controls for all.

Table S8. List of primers used for miRNAs expression analysis.

Primer	Sequence
oligodT adapter	GCGAGCACAGAATTAATACGACTCACTATAGGTTTTTTTTTTTTVN
Outer primer	GCGAGCACAGAATTAATACGACT
miR-103	AGCAGCATTGTACAGGGCTATGA
miR-26a-5p	TTCAAGTAATCCAGGATAGGCT
miR-30a-3p	CTTTCAGTCGGATGTTTGCAGC
miR-34b-5p	TAGGCAGTGTCATTAGCTGATTGAA
miR-34c-5p	AGGCAGTGTAGTTAGCTGATTGC
miR-125a-5p	TCCCTGAGACCCTTTAACCTGTGA
miR-126-5p	CATTATTACTTTTGGTACGCGAAAAA
miR-139-5p	TCTACAGTGCACGTGTCTCCAGT
miR-449c-5p	TAGGCAGTGTATTGCTAGCGGCTGT

Table S9. List of primers used for tRFs expression analysis.

Primer	Sequence
tRF-5003	GCATGGGTGGTTCAGTGGTAGAATTC
tRF-5004	GCATTGGTGGTTCAGTGGTAGAATTCTC
tRF-5007	CGCCGCTGGTGTAGTGGTATC
tRF-5008	GCGTTGGTGGTATAGTGGTGAGC
tRF-5013	GCTCGTTGGTCTAGGGGTATGATTC
tRF-5030	TCCCTGGTGGTCTAGTGGTTAGGAT
tRF-3003	TCAAATCCGGGTGCCCCCTC
tRF-3009	TCGAACCCCACTCCTGGTACC
tRF-3021	TCCCCGGCATCTCCACCA
tRF-1001	GAAGCGGGTGCTCTTATTTTAAAAAA

Table S10. List of primers used for gene expression analysis.

Gene	Forward	Reverse
ACTB	AGCGAGCATCCCCCAAAGTT	GGGCACGAAGGCTCATCATT
AGO1	GGGTATATGGGATGGAAGCGG	TCAAAGTCGACCCGTTCGTT
AGO2	GTGGAAACATTCCAGCAGGC	CCCAGAGGACGTGATAGTGC
AGO3	CCTCCATGAATTTCTGCCACTG	TTCTCTCCACAGTTTGGCCG
AGO4	TTGCTGCTTCCAAAGGTGATG	TTCCCACTTGAAGGGTAGGC
AIMP1	CGAGCCAAGTCAGCCAAGGTT	ATCTGCCTCTGCACCCTTCTGC
AIMP2	CACGCGCTACCCCCTTTTGCT	CGATGTCTTTCAGCGCCCCGT
AIMP3	GCAATCGTTCAGCAGTGGTT	ACAGATGTTGCCTGATGCCT
AKT1	ATGCAGCATCGCTTCTTTGC	TGATCATCTGGGCCGTGAAC
ARAF	GGATGGCATGAGTGTCTACGA	GGTCAGCGGGACATCTTCA
ARIH1	ACGAGGTCATCCAGAATCCA	ATCTGGCGTGTTCGAGACTT
CLP1	ACGGGAGGACCTTCTGAGTT	CCTCCACCTCAAAGCGAAGT
DICER	GTACGACTACCACAAGTACTTC	ATAGTACACCTGCCAGACTGT
DROSHA	ATCGGTTGTTCCTGAACCTGC	GGTTGTCACTCCAACGGTCT
EPRS	CAGTCCTGTTGTTTGCGAGC	TGCTGTCAACTATGGGGCAG
EIF4EBP1	TCCTTGTGCCTCCACTGATG	CTAAGGAAGGGGTGGTTGCG
EIF4E	AGGACTGAAACCACCCTCCAGCA	TCGGGGGATTAGGAGTAGGGGTGG
EIF6	TCTTCAGCAATCAGGGAGGG	ATCCCAGCAGCAATCACCTC

ELAC1	TGTGGTCCTTCGGTGTGAAG	AGAGTTCCATGGTTCGCCAG
ELAC2	GCTCACCAGTTTCCGCTGTA	GACACAAACACAGCAGCCAG
GAPDH	GAAAGCCTGCCGGTGACTAA	TGGAATTTGCCATGGGTGGA
IARS	AGGCCCCTCAGCAACAAAAT	AAACCCACTCTGGTGAGCAT
KARS	ACAGGGAACTGCGACTGGGTCA	TGGGTCCACGCTCTCTTCCTCA
MAPK13	GCATGAGAACGTCATTGGGC	CACGTAGCCAGTCATCTCGG
MAPK1	CGCCGAAGCACCATTCAAG	AGAACACCGATGTCTGAGCA
MAPK3	CTAAGGAGCGGCTGAAGGAG	GCCTCAGCAAAGGAGAGAGG
MARS	AGATTGTGTGGTCCCGTTCC	TGGTCAATGTGAGTCAGGGC
mTOR	AGCTGCCAAGTGCCGAGCAT	AGCAGCCATCCGGGCCATCTT
MYC	TCCATGAGGAGACACCGCCCA	TGGGGCTGGTGCATTTTCGGT
PABPC1	CTCCAGAGTCACTCCGTTCT	TCGGTGAAGCACAAGTTTCTT
PAQR4	GAGGTTCCGAGGCTCAAAGG	CTGGTTTCATCCGGCACTCC
POP1	TGCCTTGGGGAGAGGCCAACA	GGTTCCACACACTGGCACGCT
POP5	AGCTTGTGTGGTCAGCTCTTCCCT	AAGTGGGCCTGAAGCCTGAGCA
PTEN	GTGGAGTTTACCGGCAGCAT	CTGGCAGGTAGAAGGCAACT
QARS	ACTCCCTGTCGCTCTTCACT	GGGCTCAGTGTGGATCTTCTT
RARS	CGGTGCATTTTACGGTTCCC	ACGCCACATGTTCACCTTCA
RICTOR	TTTTCGGGGATTTCTGGATGC	GACAGCAAATGGGAAACCTCA
RPP14	TGCGCCTTGGGTAGCTTGTCCT	GCGGCATCAACCTCCCCAAACA
RPP20	TCCGTGATATGGCTCTTCGC	ACCGCATACACACACTCAGG
RPP21	TCCTGTACCAGGCCGCCCATT	TGGCAAGGGTTGTAGTGGTTTGGA
RPP25	TGACCGCGCACTAGCTGTCCT	GCCAACCCTCACAAACCGGCA
RPP29	CACCAAAGAAGACCGCCTGA	TGCGACCTTCCCATGTTGTT
RPP30	CTGAAAGTGACGCCAAGGCTGC	AAGTGACTCTCATCAGCCCAGGACA
RPP38	ACCACGGATTCGCCATCGTGAG	GCTCCAGCGGATGATGTATGGGT
RPP40	CCGCGGCACTTACTGGTTTGC	TCCCATTTGGTAGCAGGGCAACAG
RPS6	GCCCCAAAAGAGCTAGCAGA	TGCAGCAGCCTACTGAGAAC
RPS6KB	TTGGTCAGGCAGTCAACACT	TGCAGGACACGTGGAGTAAC
RPTOR	ATTCCCACCGCTCACTCATC	TTGACGCTCACACTCACGAT
SSB	AGTCGTTGCTGTTGCTGTTTG	AGGTAGGGGTTTGCTTGGAG
WARS	ATCAGGAAGGATTACACCAGCG	TGCAATGTTAGCCAGCACCA
XPO5	ATGGCAGAGCCTTTCACCAA	CACGAAGCATGGGTCTGAGT
YARS	AAGCAGGGGTAGCGGAGCCAT	TGCGTGGAGGTCCGCAAACA

Table S11. List of antibodies used for Western blot analysis.

Antibody	Catalog #	Supplier
β-Actin	4967	Cell Signaling
phospho-p38 MAPK (T180/Y182)	4631	Cell Signaling
phospho-p44/42 MAPK (T202/Y204)	4376	Cell Signaling
phospho-AKT (S473)	3586	Cell Signaling
mTOR	2983	Cell Signaling
phospho-mTOR (S2448)	5536	Cell Signaling
phospho-p70 S6 Kinase (T389)	9234	Cell Signaling
phospho-S6	4857	Cell Signaling
c-MYC	5605	Cell Signaling
4E-BP1	9644	Cell Signaling
phospho-4E-BP1 (T37/46)	2855	Cell Signaling
phospho-4E-BP1 (T70)	9455	Cell Signaling
phospho-4E-BP1 (S65)	9456	Cell Signaling
eIF4E	2067	Cell Signaling
phospho-eIF4E (S209)	9741	Cell Signaling
eIF2-alpha	9722	Cell Signaling
phospho-eIF2-alpha (S51)	9721	Cell Signaling
eEF1A	3586	Cell Signaling
eEF2	2332	Cell Signaling
anti-puromycin (12D10)	AB_2566826	Millipore

Table S12. List of siRNAs for overexpression experiments.

Table 5003.	Target Sequence
si Luc (+)	CGUACGCGGAAUACUUCGA
si Luc (−)	UCGAAGUAUUCCGCGUACG
tRF 5003b (+)	GCAUGGGUGGUUCAGUGGUAGA
tRF 5003b (−)	UCUACCACUGAACCACCCAUGC
tRF 3021a (+)	UCCCCGGCAUCUCCACCA
tRF 3021a (−)	UGGUGGAGAUGCCGGGGA

© 2020 by the authors. Licensee MDPI, Basel, Switzerland. This article is an open access article distributed under the terms and conditions of the Creative Commons Attribution (CC BY) license (http://creativecommons.org/licenses/by/4.0/).
